# Sex Differences in Comorbidity Combinations in the Swedish Population

**DOI:** 10.3390/biom12070949

**Published:** 2022-07-06

**Authors:** Laura Basso, Benjamin Boecking, Patrick Neff, Petra Brueggemann, Christopher R. Cederroth, Matthias Rose, Birgit Mazurek

**Affiliations:** 1 Tinnitus Center, Charité—Universitätsmedizin Berlin, 10117 Berlin, Germany; laura.basso@charite.de (L.B.); benjamin.boecking@charite.de (B.B.); petra.brueggemann@charite.de (P.B.); 2 Center for Neuroprosthetics, Institute of Bioengineering, École Polytechnique Fédérale de Lausanne, 1202 Geneva, Switzerland; patrick.neff@epfl.ch; 3 Department of Radiology and Medical Informatics, University of Geneva, 1211 Geneva, Switzerland; 4 Center for Cognitive Neuroscience and Department of Psychology, University of Salzburg, 5020 Salzburg, Austria; 5 Department of Psychiatry and Psychotherapy, University of Regensburg, 93053 Regensburg, Germany; 6 Laboratory of Experimental Audiology, Department of Physiology and Pharmacology, Karolinska Institutet, 17177 Stockholm, Sweden; christopher.cederroth@ki.se; 7 National Institute for Health Research (NIHR) Nottingham Biomedical Research Centre, Nottingham University Hospitals NHS Trust, Nottingham NG7 2UH, UK; 8 Hearing Sciences, Division of Clinical Neuroscience, School of Medicine, University of Nottingham, Nottingham NG7 2UH, UK; 9 Section of Psychosomatic Medicine, Medical Department, Charité—Universitätsmedizin Berlin, 10117 Berlin, Germany; matthias.rose@charite.de

**Keywords:** comorbidity, health, sex, diseases, psychiatric, digestive, musculoskeletal, skin, neurological

## Abstract

High comorbidity rates, especially mental–physical comorbidity, constitute an increasing health care burden, with women and men being differentially affected. To gain an overview of comorbidity rates stratified by sex across a range of different conditions, this study examines comorbidity patterns within and between cardiovascular, pulmonary, skin, endocrine, digestive, urogenital, musculoskeletal, neurological diseases, and psychiatric conditions. Self-report data from the LifeGene cohort of 31,825 participants from the general Swedish population (62.5% female, 18–84 years) were analyzed. Pairwise comorbidity rates of 54 self-reported conditions in women and men and adjusted odds ratios (ORs) for their comparison were calculated. Overall, the rate of pairwise disease combinations with significant comorbidity was higher in women than men (14.36% vs. 9.40%). Among psychiatric conditions, this rate was considerably high, with 41.76% in women and 39.01% in men. The highest percentages of elevated mental–physical comorbidity in women were found for musculoskeletal diseases (21.43%), digestive diseases (20.71%), and skin diseases (13.39%); in men, for musculoskeletal diseases (14.29%), neurological diseases (11.22%), and digestive diseases (10%). Implications include the need for integrating mental and physical health care services and a shift from a disease-centered to an individualized, patient-centered focus in clinical care.

## 1. Introduction

From a public health perspective, comorbidity is understood as the co-occurrence of two or more chronic medical conditions in the same individual [1,2]. Most estimates of comorbidity in primary care exceed 20% but range from 12.9 to 95.1%, depending on measurement methods and sample characteristics [3]. Individuals with comorbid conditions have more hospitalizations, greater functional decline, and increased mortality rates [4,5,6]. Co-/multimorbidity poses many challenges for clinical management, e.g., due to multi-drug therapy and possible drug-disease and drug-drug interactions [1,7,8,9]. Comorbidity is associated with increased health care costs [10] and challenges the organization of (specialized) healthcare delivery [11,12,13]. Moreover, comorbidity rates increase with age [3], and thus the associated health care burden is a growing problem in our aging societies.

For psychiatric conditions, comorbidity rates are known to be substantial [14]: 30% [15] to 40% or more [16,17] of individuals with a mental disorder (12-month prevalence) have two or more diagnoses. In addition, comorbidity and severity of psychiatric conditions are associated [16], and functional impairment and perceived unmet treatment are higher in individuals with comorbid mental disorders [15].

Another increasingly recognized problem is the high comorbidity between psychiatric conditions and physical diseases [18,19,20,21,22,23,24]. The diagnosis of comorbid psychiatric disorders in chronic physical illness can be difficult [25,26,27], and interactions between mental-physical comorbidity can negatively affect the respective treatment outcomes [24,28,29,30]. Excess mortality in people with mental illness across different diagnostic groups [31] is largely related to comorbid medical diseases [32], for which they are less likely to receive standard treatment [19,33].

The identification of frequent comorbidity patterns is important for the planning of health care organization and delivery [3,34,35,36]. Women have generally been found to have higher co-/multimorbidity rates [3,36,37,38,39,40], and sex-specific patterns have been observed [3,37,39,41,42,43]. The leading causes of disability-adjusted life-years also differ by sex [44]. This highlights the importance of investigating sex differences with regard to comorbidity patterns.

This exploratory study aims to investigate pairwise comorbidity rates of 54 self-reported conditions in women and men of the general population to identify sex-specific combinations and patterns of elevated comorbidity across a broad spectrum of conditions: comorbidity within and between cardiovascular diseases, pulmonary diseases, skin diseases, endocrine diseases, digestive diseases, urogenital diseases, musculoskeletal diseases, neurological diseases, and psychiatric conditions. The present analysis investigates pairwise comorbidity (two conditions) instead of multimorbidity to obtain an overview of comorbidity patterns—with a focus on mental–physical comorbidity—and to identify specific comorbidity combinations at the same time. Overall, generally high comorbidity of psychiatric conditions and between psychiatric conditions and physical diseases as well as sex-specific comorbidity patterns are expected.

## 2. Materials and Methods

### 2.1. Sample Description

For this study, web-based cross-sectional survey data from the LifeGene cohort collected between 2009 and 2016 in the general Swedish population were used. The recruitment process of LifeGene has been described elsewhere; inclusion criteria were residence in Sweden and recruitment-specific age criteria [45,46]. Information on the past or present occurrence of conditions was selected from the “medical history” module of the LifeGene survey. The tinnitus subsample (*N* = 7615) has been analyzed by our group in two previous studies [47,48]. 

From the available data including *N* = 31,927 participants, 102 participants were excluded from the present study due to being under the age of 18 (*N* = 68), missing age data (*N* = 2), or missing data on self-reported diseases (*N* = 32), leading to the final sample size of *N* = 31,825 participants.

Descriptive characteristics of the study sample can be found in Table 1. On average, the participants were 35.17 years old (*SD* = 11.27 years, range = 18–84 years), and 19,876 participants (62.45%) were female. This project was approved by the local ethics committee “*Regionala etikprövningsnämnden*” in Stockholm (2021-05652-01).

### 2.2. Data (LifeGene Survey)

Analyses were performed separately based on the indicated sex of the study participant in the survey (male/female). The investigated variables included the past or present occurrence of 55 different self-reported conditions overall (54 in each sex). All conditions except tinnitus were assessed by the survey question, “Which of the following diseases do you currently have or have you had?”. This question was asked twice with different response options (disease questions 1 and 2).

Disease question 1 included the following options: six ‘heart/vessel’ diseases: hypertension, hyperlipidemia, angina pectoris, myocardial infarction, cardiac arrhythmia, and thrombosis, labeled in the present study as cardiovascular diseases; two ‘pulmonary diseases’: asthma and chronic obstructive pulmonary disease (COPD); four ‘skin’ diseases: herpes, acne, eczema, and psoriasis; two ‘endocrine diseases’: diabetes and thyroid disease; ten ‘stomach’ diseases: Celiac disease, lactose intolerance, gastric acid reflux, stomach ulcer, gastritis, gallbladder problems, irritable bowel syndrome (IBS), Crohn′s disease, ulcerative colitis, and fecal incontinence, labeled in the present study as digestive diseases; and three ‘urology’ diseases: recurring urinary tract infections (UTI), prostate problems, and kidney stones, labeled in the present study as urogenital diseases. No data were missing on disease question 1.

Disease question 2 included the following options: seven ‘musculoskeletal diseases’: sciatica, chronic back pain, chronic shoulder pain, osteoarthritis, rheumatoid arthritis, systemic lupus erythematosus (SLE), and fibromyalgia; six ‘neurology’ diseases, to which bothersome tinnitus was added (see below): dyslexia, migraine, Horton′s syndrome, Meniere′s disease, epilepsy, and multiple sclerosis (MS); two ‘children and youth’ diseases: chickenpox and infectious mononucleosis (IM), which were not included in the present study; and fourteen ‘psychiatry’ diseases: burnout, depression, bipolar disease, generalized anxiety disorder/syndrome (GAD), panic disorder, agoraphobia, social anxiety, obsessive-compulsive disorder (OCD), posttraumatic stress disorder (PTSD), schizophrenia, schizoaffective disorder, Asperger′s syndrome, autism, and Tourette’s syndrome, labeled in the present study as psychiatric conditions. On disease question 2, data were missing from 6 participants.

Tinnitus was assessed by the question: “Is there a constant ringing in the ears or do you have any other bothersome sound in the ears (tinnitus)?” No data were missing on this question. Only the response option “all the time, the sound is very bothersome” was included as “bothersome tinnitus” in the analysis (as opposed to “sometimes, but the sound doesn′t bother me”), as only this category was considered to represent individuals with clinically relevant tinnitus (tinnitus disorder) [49]. Bothersome tinnitus was added to the group of neurological diseases. 

Age, education, employment, body mass index (BMI), and smoking were included as covariates in the statistical analysis. For education, the response options “don′t know/don′t want to answer” and “other” were set to missing; the variable with the remaining categories of “nine-year primary school”, “secondary school”, and “university” was treated as an ordered factor. Information on education was missing from *N* = 2959 participants. For employment, the response option “don′t know/don′t want to answer” was set to missing. The remaining options were recoded into the following categories: 1 = employed or self-employed; 2 = unemployed; 3 = on (sick or parental) leave or age pension or early retirement due to illness/disability; 4 = student; 5 = houseman/wife or other. Employment information was missing from *N* = 3574 participants. The covariate smoking was created with the categories 1 = non-smoker (never or occasionally), 2 = ex-smoker and 3 = current smoker. Information on smoking was missing from *N* = 572 participants. For the BMI, information was missing for *N* = 662 participants.

### 2.3. Statistical Analysis

The data were analyzed using R-4.0.0 [50]. Analyses were performed separately for each sex and included the comparison of pairwise comorbidity rates against the respective prevalence rates of each index condition using odds ratios (OR) obtained from age-, education-, employment-, BMI-, and smoking-adjusted logistic regression models. 

Two prevalence rates (%) were calculated (separately for women and men) for each pairwise comorbidity combination, one for each condition as the respective index condition (prevalence rate of condition A given condition B, and prevalence rate of condition B given condition A). ORs with 95% confidence intervals (CIs) were calculated based on age-, education-, employment-, BMI-, and smoking-adjusted logistic regression models (separately for women and men) to determine whether comorbidity rates were elevated in comparison to the index condition prevalence rates. ORs designate the ratio of the odds of condition A among cases with index condition B to the odds of condition A in cases without index condition B. Two ORs were calculated for each pairwise comorbidity combination, one for each condition as the respective index condition. 

For female participants, the condition ‘prostate problems’ was not applicable; for male participants, the condition ‘SLE’ was removed due to zero cases, resulting in 54 diseases for both analyses (2862 comorbidity combinations each), which led to 5724 tests in total. Correspondingly, *p*-values were adjusted for multiple testing (Bonferroni correction) with *p* < 0.05/5724 as the applied significance level.

## 3. Results

### 3.1. Sample Description

Characteristics of the study sample are summarized in Table 1.

### 3.2. Prevalence Rates

**Figure 1:**
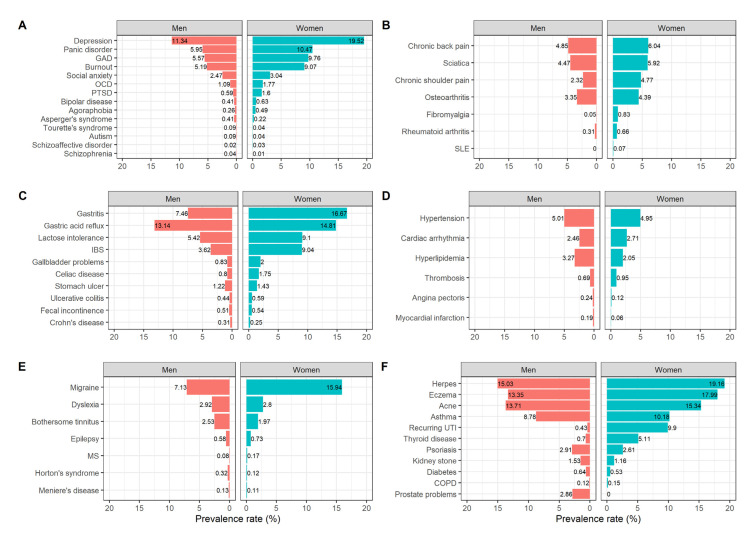
Prevalence rates (%) of psychiatric conditions (**A**), musculoskeletal diseases (**B**), digestive diseases (**C**), cardiovascular diseases (**D**), neurological diseases (**E**), and skin/pulmonary/urogenital/endocrine diseases (**F**) in male (*N* = 11,949) and female (*N* = 19,876) participants. Abbreviations: COPD = Chronic Obstructive Pulmonary Disease, IBS = Irritable Bowel Syndrome, UTI = Urinary Tract Infections, SLE = Systemic Lupus Erythematosus, MS = Multiple Sclerosis, GAD = Generalized Anxiety Disorder, OCD = Obsessive-Compulsive Disorder, PTSD = Posttraumatic Stress Disorder.

Prevalence rates of psychiatric conditions, musculoskeletal diseases, digestive diseases, cardiovascular diseases, neurological diseases, skin diseases, pulmonary diseases, urogenital diseases, and endocrine diseases in female and male participants are shown in Figure 1. The five conditions with the highest prevalence rates in women were: depression (19.52%), herpes (19.16%), eczema (17.99%), gastritis (16.67%), and migraine (15.94%); in men: herpes (15.03%), acne (13.71%), eczema (13.35%), gastric acid reflux (13.14%), and depression (11.34%).

### 3.3. Comorbidity Combinations in Female Participants

**Figure 2:**
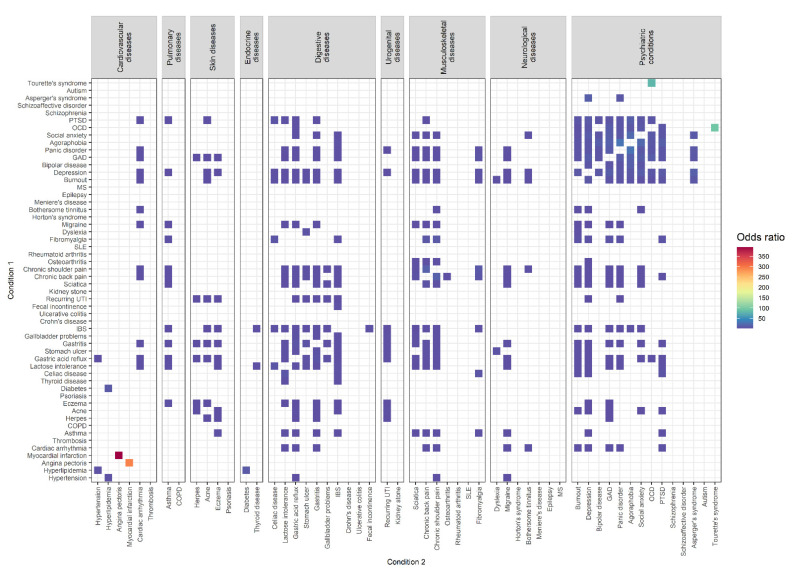
Comorbidity combinations of 54 conditions with increased odds ratios (*p* < 0.05/5724) in female participants (*N* = 19,876) based on the results of age-, education-, employment-, BMI-, and smoking-adjusted logistic regression analysis.

Comorbidity combinations with increased ORs in female participants (*N* = 19,876) can be found in Figure 2. In total, 411 of the 2862 comorbidity combinations (14.36%) had significantly increased ORs. The results of all significantly increased comorbidity combinations in female participants can be found in Supplementary Table S1.

The three combinations of comorbid conditions with the highest ORs were: angina pectoris among individuals with myocardial infarction (prevalence: 45.45%, OR = 394.14 [65.70, 2717.42])/myocardial infarction among individuals with angina pectoris (prevalence: 20.83%, OR = 293.83 [51.49, 1908.90]), Tourette’s syndrome among individuals with OCD (prevalence: 0.85%, OR = 94.38 [13.27, 658.78])/OCD among individuals with Tourette’s syndrome (prevalence: 42.86%, OR = 79.86 [12.88, 503.13]), and panic disorder among individuals with agoraphobia (prevalence: 82.65%, OR = 29.95 [16.82, 56.91])/agoraphobia among individuals with panic disorder (prevalence: 3.89%, OR = 29.61 [16.69, 56.09]).

Among diseases of the same group, elevated comorbidity combinations were found for skin diseases (50%; 6 of 12), psychiatric conditions (41.76%; 76 of 182), digestive diseases (35.56%; 32 of 90), musculoskeletal diseases (33.33%; 14 of 42), and cardiovascular diseases (13.33%; 4 of 30).

Between diseases of different groups, elevated comorbidity combinations were found for urogenital and skin diseases (37.50%; 6 of 16), musculoskeletal and pulmonary diseases (28.57%; 8 of 28), musculoskeletal and digestive diseases (25.71%, 36 of 140), urogenital and digestive diseases (25%; 10 of 40), digestive and skin diseases (22.50%; 18 of 80), psychiatric conditions and musculoskeletal diseases (21.43%; 42 of 196), psychiatric conditions and digestive diseases (20.71%; 58 of 280), digestive and pulmonary diseases (20%; 8 of 40), psychiatric conditions and skin diseases (13.39%, 15 of 112), skin and pulmonary diseases (12.50%; 2 of 16), digestive and endocrine diseases (10%; 4 of 40), endocrine and cardiovascular diseases (8.33%; 2 of 24), psychiatric conditions and neurological diseases (8.16%; 16 of 196), neurological and musculoskeletal diseases (8.16%; 8 of 98), psychiatric conditions and pulmonary diseases (7.14%, 4 of 56), psychiatric conditions and urogenital diseases (7.14%; 4 of 56), neurological and pulmonary diseases (7.14%, 2 of 28), digestive and cardiovascular diseases (6.67%; 8 of 120), neurological and cardiovascular diseases (5.95%; 5 of 84), musculoskeletal and cardiovascular diseases (5.95%; 5 of 84), psychiatric conditions and cardiovascular diseases (5.95%; 10 of 168), and neurological and digestive diseases (5.71%, 8 of 140).

Thus, the three highest percentages of elevated mental–physical comorbidity combinations were found for psychiatric conditions and musculoskeletal diseases (21.43%), psychiatric conditions and digestive diseases (20.71%), and psychiatric conditions and skin diseases (13.39%). The overall mental–physical comorbidity rate in female participants was 13.30% (149 of 1120).

### 3.4. Comorbidity Combinations in Male Participants

**Figure 3:**
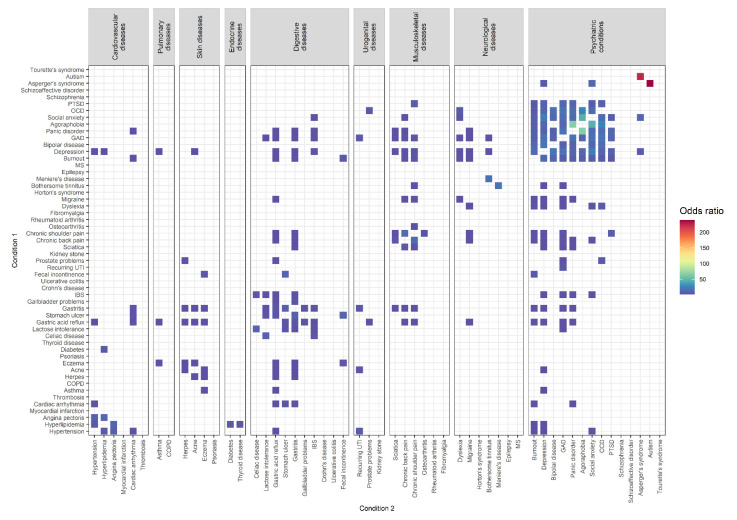
Comorbidity combinations of 54 conditions with increased odds ratios (*p* < 0.05/5724) in male participants (*N* = 11,949) based on the results of age-, education-, employment-, BMI-, and smoking-adjusted logistic regression analysis.

Comorbidity combinations with increased ORs in male participants (*N* = 11,949) can be found in Figure 3. In total, 269 of the 2862 comorbidity combinations (9.40%) had significantly increased ORs. The results of all significantly-elevated comorbidity combinations in male participants can be found in Supplementary Table S2.

The three combinations of comorbid conditions with the highest ORs were: autism among individuals with Asperger’s syndrome (prevalence: 12.24%, OR = 239.05 [20.33, 2127.42])/Asperger’s syndrome among individuals with autism (prevalence: 54.55%, OR = 224.58 [22.00, 2157.84]), panic disorder among individuals with agoraphobia (prevalence: 80.65%, OR = 66.54 [26.61, 202.10])/agoraphobia among individuals with panic disorder (prevalence: 3.52%, OR = 65.79 [26.38, 199.46]), and social anxiety among individuals with agoraphobia (prevalence: 51.61%, OR = 46.10 [20.46, 104.42])/agoraphobia among individuals with social anxiety (prevalence: 5.42%, OR = 45.43 [20.50, 101.31]).

Among diseases of the same group, elevated comorbidity combinations were found for skin diseases (50%; 6 of 12), psychiatric conditions (39.01%; 71 of 182), digestive diseases (28.89%; 26 of 90), musculoskeletal diseases (26.67%; 8 of 30), cardiovascular diseases (26.67%; 8 of 30), and neurological diseases (9.52%; 4 of 42).

Between diseases of different groups, elevated comorbidity combinations were found for skin diseases and digestive diseases (17.5%; 14 of 80), psychiatric conditions and musculoskeletal diseases (14.29%; 24 of 168), endocrine diseases and cardiovascular diseases (12.50%; 3 of 24), skin diseases and pulmonary diseases (12.5%; 2 of 16), psychiatric conditions and neurological diseases (11.22%; 22 of 196), psychiatric conditions and digestive diseases (10%; 28 of 280), musculoskeletal diseases and digestive diseases (8.33%; 10 of 120), urogenital diseases and skin diseases (8.33%; 2 of 24), digestive diseases and cardiovascular diseases (6.67%; 8 of 120), psychiatric conditions and cardiovascular diseases (6.55%; 11 of 168), psychiatric conditions and urogenital diseases (5.95%; 5 of 84), neurological diseases and musculoskeletal diseases (5.95%; 5 of 84), urogenital diseases and digestive diseases (5%; 3 of 60), digestive diseases and pulmonary diseases (5%; 2 of 40) psychiatric conditions and pulmonary diseases (3.57%; 2 of 56), urogenital diseases and cardiovascular diseases (2.78%; 1 of 36), psychiatric conditions and skin diseases (1.79%; 2 of 112), and neurological diseases and digestive diseases (1.43%; 2 of 140).

Thus, the three highest percentages of elevated mental–physical comorbidity combinations were found for psychiatric conditions and musculoskeletal diseases (14.29%), psychiatric conditions and neurological diseases (11.22%), and psychiatric conditions and digestive diseases (10%). The overall mental–physical comorbidity rate in male participants was 8.39% (94 of 1120).

## 4. Discussion

The present study investigated pairwise comorbidity rates of 54 self-reported conditions in women and men of the LifeGene cohort from the Swedish general population, including cardiovascular diseases, pulmonary diseases, skin diseases, endocrine diseases, digestive diseases, urogenital diseases, musculoskeletal diseases, neurological diseases, and psychiatric conditions. Overall, a higher percentage of significantly increased pairwise comorbidity combinations were found in women than in men (14.36% vs. 9.40%). The percentage of significantly increased pairwise comorbidity combinations between the studied psychiatric conditions was 41.76% in women and 39.01% in men. For mental–physical comorbidity, this rate was 13.30% in women and 8.39% in men. In women, the three highest percentages of elevated comorbidity with psychiatric conditions were found for musculoskeletal diseases (21.43%), digestive diseases (20.71%), and skin diseases (13.39%). For men, the three highest percentages of elevated psychiatric comorbidity were found for musculoskeletal diseases (14.29%), neurological diseases (11.22%), and digestive diseases (10%).

These findings are difficult to compare with previous studies on co-/multimorbidity [35,36,51]. However, two partially overlapping systematic reviews identified three main comorbidity patterns: comorbidity of cardiovascular and metabolic diseases, comorbidity of mental health problems (with each other or with organic diseases), and comorbidity of musculoskeletal or pain diseases (with each other or with other diseases) [3,22]. While the first pattern was less pronounced in our sample, the two other patterns were present in both sexes. Furthermore, an additional comorbidity pattern was observed here, involving digestive system disorders (with each other or with other diseases).

In line with our result, women have often been found to have higher rates of co-/multimorbidity than men [1,3,36,37,38,39,40]. A strong sex-specific finding observed here was the greatly increased comorbidity between angina and myocardial infarction in female participants (OR > 290), which was not present in men. Some findings report an increased risk for women to develop angina pectoris after myocardial infarction [52,53]. However, this relationship appears to be confounded by medical factors [53,54], which might also be possible for our observation.

The analyses were corrected for age, education, employment, BMI, and smoking. However, other factors such as environmental or socioeconomic characteristics which can increase the risk for co-/multimorbidity [1] might have influenced the results. Other limiting factors for the present analysis include the lack of temporal information and the use of self-report data as opposed to confirmed medical diagnoses, which may have introduced bias. Thus, the results need to be interpreted with caution.

### 4.1. Comorbidity between Psychiatric Conditions

As expected, the degree of comorbidity among psychiatric conditions was high, in line with the literature [14,15,16], particularly regarding the high comorbidity between depression/mood disorders and anxiety disorders [3,22,36]. In women, comorbidity between OCD and Tourette’s syndrome and between agoraphobia and panic disorder was among the three most highly elevated comorbidity combinations. In men, comorbidity between Asperger’s syndrome and autism, panic disorder and agoraphobia, and social anxiety and agoraphobia were the three most highly elevated comorbidity combinations. However, the precision of OR estimates of conditions with low prevalence rates—such as Tourette’s syndrome, agoraphobia, Asperger’s syndrome, and autism—need to be interpreted with caution.

Tourette syndrome is known to be frequently associated with obsessive-compulsive behavior or OCD [55,56], and consistent with our results, higher rates of comorbid OCD were found in females with Tourette syndrome [55]. By the newest classifications, autism and Asperger’s syndrome are not considered separate diagnoses but are conceptualized to belong to the broader group of autism spectrum disorder (ASD) [57]. In children, the prevalence of ASD is markedly higher in boys than in girls [58]. Thus, the highly increased comorbidity between Asperger’s syndrome and autism observed here in men is not surprising. The comorbidity of agoraphobia and panic disorder was high in both women and men. There is disagreement in the literature as to whether agoraphobia is indeed a frequent comorbidity of panic disorder or whether it should be conceptualized as a consequence of it [59]. However, there are indications that panic disorder can be primarily characterized as distress disorder and agoraphobia as a fear disorder [59].

### 4.2. Comorbidity between Psychiatric and Physical Conditions

#### 4.2.1. Psychiatric-Musculoskeletal Comorbidity

Comorbidity between psychiatric conditions and musculoskeletal diseases was the most common type of mental–physical comorbidity in both sexes, but with higher rates in women than men (21.43% vs. 14.29%). Respective combinations were mostly found for sciatica, chronic shoulder or back pain (both sexes), and in women, additionally for fibromyalgia (chronic, widespread musculoskeletal pain) with mood and anxiety disorders (burnout, depression, GAD, panic disorder, social anxiety, and PTSD).

In the literature, anxiety and depression have been identified as risk factors for sciatica [60,61]. Moreover, associations of fibromyalgia [62,63,64,65,66] and chronic back/shoulder pain [62,67,68,69,70,71] with mood and anxiety disorders are well-known, and interacting biopsychosocial influences appear important for their co-occurrence [72,73]. The relationship between mood/anxiety disorders and musculoskeletal pain is thought to be bidirectional [66,72,73,74,75], with both groups of conditions constituting a risk factor for the other. For the association between depression and chronic pain, predisposing genetic factors and different neurobiological mechanisms [66,72,76,77], including neuroplasticity changes [78] or neuroinflammation [79], as well as overlapping psychosocial factors [72] are assumed.

#### 4.2.2. Psychiatric-Digestive Comorbidity

The high comorbidity observed here between psychiatric conditions and diseases of the digestive system is consistent with the literature [80,81]. In men, psychiatric–digestive comorbidity (10%) was mainly present between gastric acid reflux, gastritis, and IBS with mood and anxiety disorders (burnout, depression, GAD, panic disorder, social anxiety). In women, on the other hand, more associations were present in total (20.71%). In addition to associations shared with men (of gastric acid reflux, gastritis, and IBS), they included associations of Celiac disease, lactose intolerance, and stomach ulcer with mood and anxiety disorders.

Concerning the temporal relationships of psychiatric–digestive comorbidity, affective disorders were found to precede the onset of certain digestive system diseases [80]. In contrast, in a population-based study, anxiety and depression were only found to precede the onset of functional gastrointestinal disorders in one-third of the cases, while for two-thirds, functional gastrointestinal disorders preceded the mental conditions [82]. Overall, the temporal association seems clear for some specific disease combinations, e.g., mood/anxiety disorders increase the risk for stomach ulcers [23,83,84], but for others, particularly functional gastrointestinal disorders, the relationship appears to be bidirectional. Not surprisingly, complex interactions between psychosocial factors and gastrointestinal symptoms are known for functional gastrointestinal disorders such as IBS [85,86]. Possible explanations for psychiatric-digestive comorbidity might lie in the microbiota–gut–brain axis, the bidirectional communication between the gut microbiota and the central nervous system [81]. Research on the microbiota–gut–brain axis suggests a link between gut microbiota and functional gastrointestinal disorders, such as IBS [81,86], as well as psychiatric conditions, such as depression [81,87].

#### 4.2.3. Psychiatric-Dermatological Comorbidity

In women, the comorbidity of psychiatric conditions and skin diseases (13.39%) was markedly higher than in men (1.79%). In men, the only significantly increased psychiatric-dermatological comorbidity association was found for acne and depression. In women, acne was additionally associated with burnout, GAD, and PTSD, and additional associations were found for herpes (with GAD) and eczema (with depression and GAD).

A recent meta-analysis showed significant associations of acne with depression and anxiety [88]. The influence of sex was not investigated in this meta-analysis, but there are some indications that women with acne tend to have more psychological difficulties than men [89]. High comorbidity of psychiatric conditions in patients with atopic eczema has been observed previously [90,91]. While Kauppi et al. [90] observed a higher risk for anxiety disorders in women with atopic eczema than men, but not for other psychiatric conditions, Schmitt et al. [91] found psychiatric comorbidity in atopic eczema to be independent of sex. 

Psychosocial factors may be involved in the development of skin diseases [92]. Anxiety disorders have been found to precede the onset of certain skin diseases [34]. On the other hand, skin diseases can lead to psychological problems [89,92,93]. Therefore, the relationship between psychiatric conditions and skin diseases seems to be bidirectional [92]. Overall, sex differences regarding psychiatric-dermatological comorbidity require further research.

#### 4.2.4. Psychiatric-Neurological Comorbidity

In men, neurological diseases were the third most common group with increased psychiatric comorbidity (11.22%). In women, they were the fourth most common group (8.16%). In both sexes, significantly increased comorbidity associations were found for migraine, dyslexia, and bothersome tinnitus with mood and anxiety disorders. 

The biggest sex difference was present for dyslexia. In women, it was only associated with burnout, while in men, it was additionally associated with depression, GAD, social anxiety, and OCD. Sex differences in dyslexia include higher rates in boys than girls [94], as well as potential differences in behavior, neuroanatomy, and genetics [95]. Dyslexia and emotional problems frequently co-occur [96,97]. In contrast to our results, some studies observed higher anxiety levels in dyslexic females [98,99], but further research on this topic is needed.

### 4.3. Biopsychosocial Causes for Mental–Physical Comorbidity

Overall, the observed elevated comorbidity between psychiatric conditions (mood/anxiety disorders) and physical diseases (digestive, musculoskeletal, skin, and neurological) is likely related to shared and interacting biopsychosocial influences. For example, shared predisposing genetic and environmental factors, chronic stress, immune abnormalities, and psychosocial factors seem related to fibromyalgia, IBS, and depression [77]. Overall, the effects of biological, psychological, behavioral, and social factors on mental–physical comorbidity are often interrelated [26]. Examples of shared and interacting biopsychosocial risk factors for mental-physical comorbidity, especially for functional somatic syndromes, include early life stress/chronic stress, somatization, and health-compromising behaviors.

The biological and mental consequences of early life stress and/or chronic stress experiences seem implicated in many of the identified comorbidity patterns. Stress can be associated with the onset or exacerbation of functional somatic syndromes such as IBS [86,100,101] and fibromyalgia [66,102]. For some psychiatric conditions such as PTSD and depression, stress has a central pathogenic role [103,104], but early life stress, such as childhood maltreatment, appears relevant for psychopathology in general [105,106]. Multiple adverse experiences in childhood are associated with an increased risk of developing mental health problems and major physical diseases later in life and also show associations with health-compromising behavior, e.g., problematic alcohol or drug use [107,108]. Moreover, chronic stress leads to widespread biological changes, such as persistent neuroendocrine or immune dysregulation, which increase the susceptibility to a range of physical diseases [109].

Following traumatic events, stress reactions and somatization symptoms are interlinked [110]. Somatization is a risk factor for functional somatic syndromes [111,112] and is related to higher anxiety or depression levels in these patients [113]. In addition, somatization is related to increased functional disability in primary care patients independent of psychiatric and medical comorbidity [114].

Moreover, psychiatric conditions are associated with lifestyle factors that have detrimental effects on physical health, such as smoking, heavy drinking, reduced activity, poor diet, and obesity [21,26,29,33]. In addition, socioeconomic factors, namely low education levels and living in a deprived area, have been identified as risk factors for multimorbidity [115]. Next to more direct effects such as access to medical care, lower socioeconomic status seems to negatively affect health through greater stress and more health-compromising behaviors [116]. Consequently, influences of stress, somatization, and social and behavioral factors on mental and physical health are highly interrelated.

### 4.4. Limitations

This study has some limitations. First, the cross-sectional design provides neither causal nor temporal information regarding the identified comorbidity patterns. Moreover, it is important to note that the assessed comorbidity does not necessarily mean that the conditions were present in an individual at the same time, as the survey question did not differentiate between past and present occurrences. A second limitation concerns the data source. The use of self-report disease data without medical verification of the diagnoses by health care professionals can question their reliability. However, even though the correspondence between self-report data and health administrative data is generally low, it also varies strongly and is high for some diseases, e.g., for diabetes and hypertension [117,118]. Comorbidity or multimorbidity estimates derived from self-report are often higher than those from health administrative data [117,119], but self-report data might be more sensitive for the detection of symptoms-based conditions [119]. Third, a recall bias or a bias caused by psychological influences on response tendencies could be present, e.g., due to somatization. Fourth, a selection bias regarding the sample or the included conditions cannot be excluded. Information on other diseases, such as different cancer types, was not available for the analysis but would have constituted important variables of interest [120,121]. Fifth, the analyses were performed separately based on the indicated sex, but information on sexual orientation was missing. However, multimorbidity was found to be increased among sexual minorities [122,123], and respective effects could not be considered in the present study. Finally, this study focused on the comorbidity of two conditions, although multimorbidity is a common phenomenon for many patients. Therefore, the observed bivariate associations between conditions do not necessarily reflect direct associations, as they could be caused by shared comorbidity with other conditions. However, the bivariate approach allowed us to quantify the comorbidity rates of specific disease combinations, which would not have been possible with broader multimorbidity clusters.

### 4.5. Implications

Research into shared and modifiable risk factors of frequent (sex-specific) comorbidity patterns is important for prevention and improving clinical care. Based on the high observed mental–physical comorbidity, the need for better integration of mental and physical health care services is apparent, as discussed by others [28,29,33]. Interacting effects between comorbid conditions on the respective treatment and prognosis can render the clinical management of patients with co-/multimorbidity more complex [2,33,124]. Therefore, clinical guidelines are often not applicable to patients with multiple comorbid conditions [2,125,126]. Consequently, for the clinical assessment and management of these patients, a shift from the current disease-oriented approach to a more patient-centered, individualized approach is needed [2,125,126,127]. A patient-centered approach for co-/multimorbidity management should take a holistic, biopsychosocial perspective [126], integrate and coordinate the delivery of health care across disciplines [125,127], and involve shared decision making in tailoring treatment to the individual [125,127]. Different initiatives to increase patient-centredness in co-/multimorbidity care are currently being applied, which could reflect a possible beginning in this needed transition [127].

## 5. Conclusions

The present study investigated pairwise comorbidity rates of 54 self-reported conditions in women and men of the LifeGene cohort from the Swedish general population adjusted for age, education, employment, BMI, and smoking. Overall, a higher percentage of significantly increased pairwise comorbidity combinations were found in women than in men (14.36% vs. 9.40%). The comorbidity between psychiatric conditions was considerably high in both women and men (41.76% vs. 39.01%), and highest between OCD and Tourette’s syndrome (women only), Asperger’s syndrome and autism (men only), agoraphobia and panic disorder (both), social anxiety and agoraphobia (both). For mental–physical comorbidity (women: 13.30%; men: 8.39%), the three groups with the highest elevated psychiatric comorbidity in women were musculoskeletal (21.43%), digestive (20.71%), and skin diseases (13.39%); in men, musculoskeletal (14.29%), neurological (11.22%), and digestive diseases (10%). Overlap and interactions between biopsychosocial risk factors appear relevant for the identified mental–physical comorbidity patterns, including, but not limited to, influences of chronic or early life stress, somatization, and health-compromising behavior. Limitations of the present study included the absence of any temporal information and the use of self-report data. The results implicate the need for a more integrated mental–physical health care approach and improved patient-centered treatment.

## Figures and Tables

**Table 1:** Sample characteristics (*N* = 31,825).

Variable	M/N	SD/%
Age	35.17	11.27
Sex		
*Female*	19,876	62.45
*Male*	11,949	37.55
Marital status		
*Married*	8047	25.29
*Cohabiting*	11,098	34.87
*Single*	7585	23.83
*Separated/divorced*	1223	3.84
*Living apart*	3033	9.53
*Widowed*	111	0.35
*Same-sex marriage*	14	0.04
*Missing*	714	2.24
Education ^1^		
*9-year primary school*	612	1.92
*Secondary school*	7167	22.52
*University*	21,087	66.26
*Missing*	2959	9.30
Employment status		
*Employed*	18,097	56.86
*Unemployed*	750	2.36
*Self-employed ^2^*	2325	7.31
*Age pension*	703	2.21
*Early retirement ^3^*	167	0.52
*Sick leave*	165	0.52
*Parental leave*	916	2.88
*Student*	4626	14.54
*On leave*	48	0.15
*Housewife/man*	64	0.20
*Other*	390	1.23
*Missing*	3574	11.23
Smoking		
*Never*	20,600	64.73
*Ex-smoker*	8350	26.24
*Current smoker*	2303	7.24
*Missing*	572	1.80
BMI (*N* = 31,163)	20.87	2.94

^1^ Highest or current education level. ^2^ Running owned or part-owned company. ^3^ Early retirement due to illness/disability (activity or sickness benefit). Abbreviations: M = mean; *N* = number of cases; SD = standard deviation.

## Data Availability

The data presented in this study are available from Nancy Pedersen (nancy.pedersen@ki.se), Department of Medical Epidemiology and Biostatistics, Karolinska Institutet, Stockholm, Sweden, but restrictions apply to the availability of these data: restrictions are based on the Swedish Act (2013:794) requiring that a valid ethical approval is obtained in Sweden.

## Supplementary Materials

The following supporting information can be downloaded at: https://www.mdpi.com/article/10.3390/biom12070949/s1, Table S1: Significantly elevated comorbidity combinations (*p* < 0.05/5724) in female participants (N = 19,876) sorted by odds ratio; Table S2: Significantly elevated comorbidity combinations (*p* < 0.05/5724) in male participants (N = 11,960) sorted by odds ratio.
