# Supplementary material for: Sex Differences in Comorbidity Combinations in the Swedish Population
Source: Biomolecules. 2022 Jul 6;12(7):949. doi: 10.3390/biom12070949 (PMC9313065; doi:10.3390/biom12070949)
Supplement: Supplementary file 1 [file biomolecules-12-00949-s001.zip › Table S1.pdf]

Table S1. Significantly elevated comorbidity combinations (p<0.05/5724) in female participants (N=19876) sorted by odds ratio.

Group of Condition 1	Group of Condition 2	Condition 1 …	… in individuals with Condition 2	Prevalence (%)	Odds ratio	Lower 95%-CI	Upper 95%-CI
Cardiovascular diseases	Cardiovascular diseases	Angina pectoris	Myocardial infarction	45.455	394.135	65.704	2717.418
Cardiovascular diseases	Cardiovascular diseases	Myocardial infarction	Angina pectoris	20.833	293.828	51.485	1908.896
Psychiatric conditions	Psychiatric conditions	Tourette’s syndrome	OCD	0.855	94.384	13.271	658.776
Psychiatric conditions	Psychiatric conditions	OCD	Tourette’s syndrome	42.857	79.857	12.882	503.130
Psychiatric conditions	Psychiatric conditions	Panic disorder	Agoraphobia	82.653	29.954	16.817	56.907
Psychiatric conditions	Psychiatric conditions	Agoraphobia	Panic disorder	3.890	29.612	16.686	56.087
Psychiatric conditions	Psychiatric conditions	Agoraphobia	Social anxiety	8.609	21.175	12.606	35.237
Psychiatric conditions	Psychiatric conditions	Social anxiety	Agoraphobia	53.061	20.960	12.421	35.018
Musculoskeletal diseases	Musculoskeletal diseases	Chronic shoulder pain	Chronic back pain	34.055	16.074	13.510	19.117
Musculoskeletal diseases	Musculoskeletal diseases	Chronic back pain	Chronic shoulder pain	43.098	16.058	13.500	19.094
Psychiatric conditions	Psychiatric conditions	Asperger’s syndrome	Depression	0.876	14.354	5.705	43.681
Psychiatric conditions	Psychiatric conditions	Agoraphobia	GAD	3.507	14.024	8.443	23.754
Psychiatric conditions	Psychiatric conditions	GAD	Agoraphobia	69.388	13.801	8.271	23.485
Psychiatric conditions	Psychiatric conditions	Depression	Asperger’s syndrome	77.273	13.335	5.268	40.718
Psychiatric conditions	Psychiatric conditions	Agoraphobia	PTSD	7.210	13.267	6.967	24.015
Psychiatric conditions	Psychiatric conditions	PTSD	Agoraphobia	23.469	13.259	6.953	24.031
Psychiatric conditions	Psychiatric conditions	Agoraphobia	Bipolar disease	11.111	12.716	5.122	28.098
Psychiatric conditions	Psychiatric conditions	Bipolar disease	Agoraphobia	14.286	12.575	5.062	27.792
Psychiatric conditions	Psychiatric conditions	Depression	GAD	68.231	11.942	10.545	13.542
Psychiatric conditions	Psychiatric conditions	GAD	Depression	34.098	11.876	10.489	13.464
Psychiatric conditions	Psychiatric conditions	Social anxiety	GAD	17.225	11.808	9.598	14.536
Psychiatric conditions	Psychiatric conditions	GAD	Social anxiety	55.298	11.767	9.563	14.490
Psychiatric conditions	Psychiatric conditions	Panic disorder	GAD	47.808	10.997	9.661	12.520
Psychiatric conditions	Psychiatric conditions	GAD	Panic disorder	44.524	10.986	9.653	12.505
Psychiatric conditions	Psychiatric conditions	Social anxiety	Depression	11.211	10.674	8.606	13.316
Psychiatric conditions	Psychiatric conditions	Depression	Social anxiety	72.020	10.627	8.568	13.259
Psychiatric conditions	Psychiatric conditions	GAD	OCD	58.974	10.531	8.029	13.841
Psychiatric conditions	Psychiatric conditions	OCD	GAD	10.676	10.524	8.024	13.831
Endocrine diseases	Cardiovascular diseases	Diabetes	Hyperlipidemia	5.882	9.533	5.146	16.996
Musculoskeletal diseases	Musculoskeletal diseases	Chronic shoulder pain	Fibromyalgia	38.788	9.314	6.296	13.625
Cardiovascular diseases	Endocrine diseases	Hyperlipidemia	Diabetes	22.857	9.199	4.965	16.451
Psychiatric conditions	Psychiatric conditions	PTSD	Depression	5.825	9.085	6.834	12.196
Psychiatric conditions	Psychiatric conditions	Depression	PTSD	70.846	9.052	6.803	12.161
Psychiatric conditions	Psychiatric conditions	Social anxiety	OCD	26.211	8.991	6.390	12.476
Psychiatric conditions	Psychiatric conditions	Asperger’s syndrome	Social anxiety	2.318	8.839	3.310	21.250
Psychiatric conditions	Psychiatric conditions	OCD	Social anxiety	15.232	8.824	6.287	12.215
Musculoskeletal diseases	Musculoskeletal diseases	Fibromyalgia	Chronic shoulder pain	6.744	8.723	5.891	12.773
Psychiatric conditions	Psychiatric conditions	Agoraphobia	OCD	5.128	8.302	3.905	16.267
Psychiatric conditions	Psychiatric conditions	OCD	Agoraphobia	18.367	8.067	3.810	15.731
Psychiatric conditions	Psychiatric conditions	Depression	Burnout	58.491	8.061	7.143	9.104
Psychiatric conditions	Psychiatric conditions	Burnout	Depression	27.165	7.956	7.053	8.981
Musculoskeletal diseases	Musculoskeletal diseases	Chronic back pain	Fibromyalgia	43.636	7.892	5.404	11.421
Psychiatric conditions	Psychiatric conditions	Asperger’s syndrome	Panic disorder	0.865	7.856	3.447	18.000
Psychiatric conditions	Psychiatric conditions	Social anxiety	Panic disorder	13.785	7.712	6.279	9.463
Psychiatric conditions	Psychiatric conditions	Panic disorder	Social anxiety	47.517	7.683	6.254	9.430
Psychiatric conditions	Psychiatric conditions	Asperger’s syndrome	Burnout	0.777	7.682	3.335	17.258
Psychiatric conditions	Psychiatric conditions	Agoraphobia	Depression	1.830	7.631	4.580	13.135
Psychiatric conditions	Psychiatric conditions	Panic disorder	Asperger’s syndrome	40.909	7.582	3.300	17.474

Group of Condition 1	Group of Condition 2	Condition 1 …	… in individuals with Condition 2	Prevalence (%)	Odds ratio	Lower 95%-CI	Upper 95%-CI
Psychiatric conditions	Psychiatric conditions	Bipolar disease	Depression	2.191	7.569	4.798	12.199
Digestive diseases	Digestive diseases	Celiac disease	Lactose intolerance	7.463	7.516	5.759	9.772
Psychiatric conditions	Psychiatric conditions	Depression	Agoraphobia	72.449	7.509	4.490	12.966
Digestive diseases	Digestive diseases	Lactose intolerance	Celiac disease	38.793	7.502	5.749	9.753
Psychiatric conditions	Psychiatric conditions	Panic disorder	Depression	31.572	7.467	6.657	8.380
Psychiatric conditions	Psychiatric conditions	Depression	Bipolar disease	67.460	7.459	4.727	12.027
Psychiatric conditions	Psychiatric conditions	Depression	Panic disorder	58.838	7.455	6.646	8.367
Psychiatric conditions	Psychiatric conditions	PTSD	Panic disorder	7.061	7.433	5.629	9.796
Psychiatric conditions	Psychiatric conditions	Panic disorder	PTSD	46.082	7.396	5.600	9.750
Musculoskeletal diseases	Musculoskeletal diseases	Fibromyalgia	Chronic back pain	5.995	7.268	4.961	10.550
Psychiatric conditions	Psychiatric conditions	OCD	Panic disorder	8.838	7.224	5.517	9.452
Psychiatric conditions	Psychiatric conditions	Panic disorder	OCD	52.422	7.223	5.510	9.459
Psychiatric conditions	Psychiatric conditions	PTSD	Social anxiety	10.099	6.953	4.798	9.875
Psychiatric conditions	Psychiatric conditions	Social anxiety	PTSD	19.122	6.862	4.726	9.763
Psychiatric conditions	Psychiatric conditions	Asperger’s syndrome	GAD	1.031	6.827	2.938	15.700
Psychiatric conditions	Psychiatric conditions	Depression	OCD	65.242	6.746	5.140	8.906
Psychiatric conditions	Psychiatric conditions	OCD	PTSD	10.031	6.704	4.157	10.411
Psychiatric conditions	Psychiatric conditions	OCD	Depression	5.902	6.698	5.110	8.831
Psychiatric conditions	Psychiatric conditions	PTSD	OCD	9.117	6.676	4.112	10.445
Psychiatric conditions	Psychiatric conditions	PTSD	GAD	7.272	6.536	4.908	8.670
Psychiatric conditions	Psychiatric conditions	GAD	PTSD	44.201	6.461	4.847	8.579
Psychiatric conditions	Psychiatric conditions	Bipolar disease	GAD	3.094	6.345	3.980	10.031
Psychiatric conditions	Psychiatric conditions	GAD	Bipolar disease	47.619	6.272	3.952	9.872
Psychiatric conditions	Psychiatric conditions	Bipolar disease	Social anxiety	4.305	6.137	3.340	10.658
Psychiatric conditions	Psychiatric conditions	Social anxiety	Bipolar disease	20.635	6.071	3.293	10.573
Psychiatric conditions	Psychiatric conditions	Agoraphobia	Burnout	2.164	6.026	3.609	9.905
Psychiatric conditions	Psychiatric conditions	Burnout	Agoraphobia	39.796	5.918	3.527	9.778
Digestive diseases	Musculoskeletal diseases	Celiac disease	Fibromyalgia	6.061	5.860	2.683	11.341
Musculoskeletal diseases	Digestive diseases	Fibromyalgia	Celiac disease	2.874	5.646	2.574	10.996
Psychiatric conditions	Psychiatric conditions	PTSD	Burnout	6.437	5.397	4.052	7.142
Psychiatric conditions	Psychiatric conditions	Bipolar disease	PTSD	3.762	5.377	2.421	10.638
Psychiatric conditions	Psychiatric conditions	Burnout	GAD	26.044	5.322	4.601	6.149
Psychiatric conditions	Psychiatric conditions	Burnout	Panic disorder	26.177	5.320	4.629	6.109
Psychiatric conditions	Psychiatric conditions	Burnout	PTSD	36.364	5.295	3.972	7.014
Psychiatric conditions	Psychiatric conditions	Panic disorder	Burnout	30.244	5.257	4.578	6.031
Psychiatric conditions	Psychiatric conditions	GAD	Burnout	28.024	5.178	4.483	5.975
Psychiatric conditions	Musculoskeletal diseases	GAD	Fibromyalgia	27.273	5.002	3.216	7.633
Psychiatric conditions	Musculoskeletal diseases	PTSD	Fibromyalgia	9.091	4.940	2.331	9.418
Digestive diseases	Digestive diseases	Gastric acid reflux	Gastritis	36.342	4.922	4.456	5.437
Digestive diseases	Digestive diseases	Gastritis	Gastric acid reflux	40.897	4.922	4.455	5.437
Musculoskeletal diseases	Psychiatric conditions	Fibromyalgia	GAD	2.321	4.838	3.138	7.301
Digestive diseases	Psychiatric conditions	Celiac disease	PTSD	6.270	4.824	2.729	7.969
Psychiatric conditions	Digestive diseases	PTSD	Celiac disease	5.747	4.779	2.700	7.905
Psychiatric conditions	Psychiatric conditions	Bipolar disease	Panic disorder	2.546	4.680	2.918	7.394
Psychiatric conditions	Psychiatric conditions	Panic disorder	Bipolar disease	42.063	4.675	2.923	7.365
Musculoskeletal diseases	Musculoskeletal diseases	Chronic back pain	Sciatica	22.175	4.608	3.847	5.501
Musculoskeletal diseases	Musculoskeletal diseases	Sciatica	Chronic back pain	21.732	4.582	3.824	5.471
Psychiatric conditions	Musculoskeletal diseases	Depression	Fibromyalgia	49.697	4.534	3.140	6.544
Digestive diseases	Digestive diseases	Stomach ulcer	Gastritis	4.075	4.532	3.414	6.008
Digestive diseases	Digestive diseases	Gastritis	Stomach ulcer	47.368	4.524	3.408	5.998

Group of Condition 1	Group of Condition 2	Condition 1 …	… in individuals with Condition 2	Prevalence (%)	Odds ratio	Lower 95%-CI	Upper 95%-CI
Neurological diseases	Digestive diseases	Dyslexia	Stomach ulcer	9.825	4.378	2.655	6.868
Digestive diseases	Neurological diseases	Stomach ulcer	Dyslexia	5.036	4.346	2.634	6.821
Psychiatric conditions	Musculoskeletal diseases	Burnout	Fibromyalgia	34.545	4.050	2.730	5.914
Musculoskeletal diseases	Psychiatric conditions	Fibromyalgia	Depression	2.113	3.991	2.781	5.719
Psychiatric conditions	Musculoskeletal diseases	Panic disorder	Fibromyalgia	27.879	3.968	2.577	5.986
Digestive diseases	Musculoskeletal diseases	IBS	Fibromyalgia	29.091	3.821	2.510	5.690
Digestive diseases	Digestive diseases	Gastric acid reflux	Stomach ulcer	39.298	3.780	2.821	5.037
Musculoskeletal diseases	Digestive diseases	Fibromyalgia	IBS	2.674	3.769	2.480	5.600
Digestive diseases	Digestive diseases	Stomach ulcer	Gastric acid reflux	3.804	3.759	2.804	5.010
Musculoskeletal diseases	Psychiatric conditions	Fibromyalgia	Panic disorder	2.209	3.744	2.443	5.614
Psychiatric conditions	Digestive diseases	Agoraphobia	IBS	1.671	3.677	2.096	6.197
Cardiovascular diseases	Cardiovascular diseases	Hyperlipidemia	Hypertension	11.699	3.654	2.759	4.804
Digestive diseases	Digestive diseases	Fecal incontinence	IBS	1.670	3.629	2.114	5.983
Cardiovascular diseases	Cardiovascular diseases	Hypertension	Hyperlipidemia	28.186	3.629	2.740	4.772
Digestive diseases	Digestive diseases	IBS	Fecal incontinence	28.037	3.620	2.109	5.971
Digestive diseases	Psychiatric conditions	IBS	Agoraphobia	30.612	3.550	2.018	5.999
Musculoskeletal diseases	Psychiatric conditions	Fibromyalgia	Burnout	3.163	3.457	2.322	5.065
Psychiatric conditions	Psychiatric conditions	Social anxiety	Burnout	8.158	3.438	2.708	4.333
Psychiatric conditions	Psychiatric conditions	Burnout	Social anxiety	24.338	3.423	2.692	4.320
Digestive diseases	Digestive diseases	IBS	Lactose intolerance	19.900	3.180	2.750	3.668
Digestive diseases	Digestive diseases	Lactose intolerance	IBS	20.045	3.163	2.735	3.649
Psychiatric conditions	Cardiovascular diseases	PTSD	Cardiac arrhythmia	5.214	3.135	1.863	4.983
Cardiovascular diseases	Psychiatric conditions	Cardiac arrhythmia	PTSD	8.777	3.096	1.837	4.931
Musculoskeletal diseases	Musculoskeletal diseases	Chronic shoulder pain	Sciatica	13.509	3.034	2.439	3.747
Musculoskeletal diseases	Musculoskeletal diseases	Sciatica	Chronic shoulder pain	16.754	3.019	2.428	3.728
Psychiatric conditions	Psychiatric conditions	Burnout	OCD	21.368	2.977	2.111	4.123
Digestive diseases	Digestive diseases	Lactose intolerance	Stomach ulcer	24.561	2.959	2.083	4.124
Digestive diseases	Musculoskeletal diseases	Stomach ulcer	Chronic shoulder pain	4.215	2.958	1.901	4.432
Psychiatric conditions	Neurological diseases	Social anxiety	Bothersome tinnitus	6.122	2.955	1.825	4.557
Digestive diseases	Digestive diseases	Stomach ulcer	Lactose intolerance	3.870	2.951	2.077	4.113
Pulmonary diseases	Musculoskeletal diseases	Asthma	Fibromyalgia	20.000	2.937	1.869	4.479
Psychiatric conditions	Musculoskeletal diseases	Burnout	Chronic shoulder pain	22.972	2.921	2.408	3.524
Musculoskeletal diseases	Digestive diseases	Chronic shoulder pain	Stomach ulcer	14.035	2.919	1.875	4.376
Musculoskeletal diseases	Neurological diseases	Chronic shoulder pain	Bothersome tinnitus	14.031	2.897	2.012	4.070
Musculoskeletal diseases	Psychiatric conditions	Chronic shoulder pain	Burnout	12.098	2.888	2.379	3.488
Neurological diseases	Musculoskeletal diseases	Bothersome tinnitus	Chronic shoulder pain	5.796	2.880	1.999	4.048
Neurological diseases	Psychiatric conditions	Bothersome tinnitus	Social anxiety	3.974	2.849	1.758	4.397
Psychiatric conditions	Psychiatric conditions	OCD	Burnout	4.162	2.832	2.016	3.905
Psychiatric conditions	Digestive diseases	PTSD	Lactose intolerance	3.927	2.792	1.995	3.836
Musculoskeletal diseases	Pulmonary diseases	Fibromyalgia	Asthma	1.630	2.762	1.763	4.195
Digestive diseases	Psychiatric conditions	Lactose intolerance	PTSD	22.257	2.758	1.970	3.793
Cardiovascular diseases	Neurological diseases	Cardiac arrhythmia	Bothersome tinnitus	8.418	2.754	1.730	4.193
Neurological diseases	Cardiovascular diseases	Bothersome tinnitus	Cardiac arrhythmia	6.134	2.742	1.723	4.175
Psychiatric conditions	Digestive diseases	Burnout	Celiac disease	18.391	2.726	1.953	3.735
Digestive diseases	Psychiatric conditions	Celiac disease	Burnout	3.552	2.720	1.950	3.725
Digestive diseases	Musculoskeletal diseases	Stomach ulcer	Chronic back pain	3.497	2.690	1.779	3.937
Digestive diseases	Urogenital diseases	Gallbladder problems	Recurring UTI	3.915	2.665	1.991	3.517
Skin diseases	Pulmonary diseases	Eczema	Asthma	33.893	2.665	2.375	2.987
Pulmonary diseases	Skin diseases	Asthma	Eczema	19.189	2.664	2.374	2.987
Urogenital diseases	Digestive diseases	Recurring UTI	Gallbladder problems	19.395	2.658	1.986	3.507

Group of Condition 1	Group of Condition 2	Condition 1 …	… in individuals with Condition 2	Prevalence (%)	Odds ratio	Lower 95%-CI	Upper 95%-CI
Musculoskeletal diseases	Digestive diseases	Chronic back pain	Stomach ulcer	14.737	2.657	1.756	3.894
Digestive diseases	Digestive diseases	IBS	Celiac disease	20.977	2.653	1.924	3.594
Digestive diseases	Digestive diseases	Celiac disease	IBS	4.065	2.650	1.922	3.591
Digestive diseases	Digestive diseases	Gastric acid reflux	Gallbladder problems	32.242	2.630	2.053	3.346
Digestive diseases	Digestive diseases	Gallbladder problems	Gastric acid reflux	4.348	2.620	2.046	3.332
Digestive diseases	Digestive diseases	IBS	Gastritis	17.326	2.609	2.309	2.944
Digestive diseases	Digestive diseases	Gastritis	IBS	31.960	2.605	2.305	2.939
Musculoskeletal diseases	Cardiovascular diseases	Chronic shoulder pain	Cardiac arrhythmia	11.173	2.542	1.824	3.467
Cardiovascular diseases	Musculoskeletal diseases	Cardiac arrhythmia	Chronic shoulder pain	6.322	2.535	1.819	3.459
Psychiatric conditions	Musculoskeletal diseases	GAD	Chronic shoulder pain	20.548	2.523	2.043	3.097
Musculoskeletal diseases	Psychiatric conditions	Chronic shoulder pain	GAD	10.057	2.517	2.037	3.091
Psychiatric conditions	Cardiovascular diseases	GAD	Cardiac arrhythmia	18.436	2.497	1.884	3.266
Cardiovascular diseases	Psychiatric conditions	Cardiac arrhythmia	GAD	5.106	2.495	1.884	3.261
Digestive diseases	Psychiatric conditions	Stomach ulcer	Burnout	3.385	2.494	1.729	3.519
Psychiatric conditions	Musculoskeletal diseases	Social anxiety	Sciatica	5.607	2.488	1.818	3.342
Psychiatric conditions	Digestive diseases	Burnout	Stomach ulcer	21.404	2.482	1.721	3.500
Psychiatric conditions	Musculoskeletal diseases	PTSD	Chronic back pain	4.163	2.469	1.666	3.554
Psychiatric conditions	Musculoskeletal diseases	Depression	Chronic shoulder pain	36.038	2.464	2.087	2.903
Psychiatric conditions	Musculoskeletal diseases	Social anxiety	Chronic shoulder pain	6.428	2.430	1.728	3.338
Digestive diseases	Digestive diseases	IBS	Gastric acid reflux	16.780	2.428	2.133	2.758
Musculoskeletal diseases	Psychiatric conditions	Sciatica	Social anxiety	10.927	2.426	1.769	3.267
Digestive diseases	Digestive diseases	Gastric acid reflux	IBS	27.506	2.426	2.132	2.756
Digestive diseases	Musculoskeletal diseases	Gallbladder problems	Chronic shoulder pain	4.953	2.418	1.670	3.407
Musculoskeletal diseases	Psychiatric conditions	Chronic shoulder pain	Depression	8.814	2.416	2.046	2.846
Musculoskeletal diseases	Digestive diseases	Chronic shoulder pain	Gallbladder problems	11.839	2.408	1.664	3.393
Musculoskeletal diseases	Psychiatric conditions	Chronic back pain	PTSD	15.674	2.405	1.620	3.466
Digestive diseases	Digestive diseases	Gallbladder problems	Gastritis	3.682	2.405	1.880	3.057
Digestive diseases	Digestive diseases	Gastritis	Gallbladder problems	30.730	2.397	1.872	3.048
Musculoskeletal diseases	Psychiatric conditions	Chronic shoulder pain	Social anxiety	10.099	2.392	1.699	3.290
Psychiatric conditions	Digestive diseases	PTSD	Gastritis	3.231	2.359	1.772	3.113
Digestive diseases	Psychiatric conditions	Gastritis	PTSD	33.542	2.334	1.753	3.082
Musculoskeletal diseases	Psychiatric conditions	Chronic back pain	GAD	11.707	2.329	1.921	2.808
Psychiatric conditions	Musculoskeletal diseases	GAD	Chronic back pain	18.901	2.329	1.923	2.807
Psychiatric conditions	Digestive diseases	Depression	Gastritis	32.005	2.307	2.094	2.541
Digestive diseases	Psychiatric conditions	Gastritis	Depression	27.320	2.307	2.094	2.541
Psychiatric conditions	Musculoskeletal diseases	Burnout	Chronic back pain	19.234	2.306	1.923	2.753
Digestive diseases	Digestive diseases	IBS	Stomach ulcer	20.702	2.298	1.583	3.252
Cardiovascular diseases	Musculoskeletal diseases	Cardiac arrhythmia	Chronic back pain	5.912	2.291	1.687	3.056
Musculoskeletal diseases	Cardiovascular diseases	Chronic back pain	Cardiac arrhythmia	13.222	2.290	1.689	3.052
Digestive diseases	Psychiatric conditions	Gastritis	Burnout	29.079	2.289	2.014	2.597
Digestive diseases	Psychiatric conditions	Gastritis	Panic disorder	29.827	2.287	2.024	2.581
Musculoskeletal diseases	Psychiatric conditions	Chronic back pain	Burnout	12.819	2.283	1.903	2.727
Digestive diseases	Digestive diseases	Stomach ulcer	IBS	3.285	2.282	1.572	3.231
Psychiatric conditions	Digestive diseases	Burnout	Gastritis	15.821	2.279	2.005	2.586
Psychiatric conditions	Digestive diseases	Panic disorder	Gastritis	18.750	2.278	2.016	2.571
Urogenital diseases	Digestive diseases	Recurring UTI	Stomach ulcer	20.351	2.276	1.584	3.199
Digestive diseases	Psychiatric conditions	IBS	Panic disorder	16.330	2.273	1.953	2.636
Digestive diseases	Psychiatric conditions	Gastritis	GAD	30.015	2.273	2.002	2.577
Digestive diseases	Urogenital diseases	Stomach ulcer	Recurring UTI	2.949	2.269	1.578	3.190
Urogenital diseases	Digestive diseases	Recurring UTI	Gastritis	17.688	2.265	2.006	2.554

Group of Condition 1	Group of Condition 2	Condition 1 …	… in individuals with Condition 2	Prevalence (%)	Odds ratio	Lower 95%-CI	Upper 95%-CI
Digestive diseases	Musculoskeletal diseases	IBS	Chronic shoulder pain	17.703	2.263	1.838	2.766
Psychiatric conditions	Musculoskeletal diseases	Social anxiety	Chronic back pain	6.162	2.262	1.656	3.031
Psychiatric conditions	Digestive diseases	Panic disorder	IBS	18.942	2.260	1.942	2.622
Digestive diseases	Urogenital diseases	Gastritis	Recurring UTI	29.792	2.259	2.001	2.547
Digestive diseases	Musculoskeletal diseases	Gallbladder problems	Sciatica	5.268	2.255	1.631	3.061
Psychiatric conditions	Digestive diseases	GAD	Gastritis	17.572	2.255	1.986	2.556
Musculoskeletal diseases	Digestive diseases	Chronic shoulder pain	IBS	9.359	2.253	1.830	2.754
Psychiatric conditions	Neurological diseases	Burnout	Bothersome tinnitus	19.643	2.252	1.658	3.013
Psychiatric conditions	Musculoskeletal diseases	Depression	Chronic back pain	34.804	2.243	1.932	2.599
Psychiatric conditions	Cardiovascular diseases	Panic disorder	Cardiac arrhythmia	18.436	2.238	1.700	2.909
Cardiovascular diseases	Psychiatric conditions	Cardiac arrhythmia	Panic disorder	4.755	2.237	1.698	2.908
Digestive diseases	Psychiatric conditions	IBS	Depression	15.335	2.234	1.978	2.521
Musculoskeletal diseases	Psychiatric conditions	Chronic back pain	Social anxiety	12.252	2.233	1.633	2.996
Psychiatric conditions	Digestive diseases	Depression	IBS	33.148	2.228	1.972	2.515
Neurological diseases	Musculoskeletal diseases	Migraine	Chronic shoulder pain	27.713	2.216	1.861	2.630
Musculoskeletal diseases	Psychiatric conditions	Chronic back pain	Depression	10.773	2.214	1.907	2.564
Digestive diseases	Psychiatric conditions	Gastric acid reflux	Panic disorder	26.945	2.212	1.945	2.511
Psychiatric conditions	Digestive diseases	Panic disorder	Gastric acid reflux	19.069	2.210	1.944	2.509
Musculoskeletal diseases	Digestive diseases	Sciatica	Gallbladder problems	15.617	2.208	1.594	3.003
Musculoskeletal diseases	Neurological diseases	Chronic shoulder pain	Migraine	8.302	2.206	1.852	2.618
Digestive diseases	Digestive diseases	Gallbladder problems	IBS	3.842	2.205	1.620	2.949
Psychiatric conditions	Pulmonary diseases	PTSD	Asthma	3.014	2.197	1.552	3.045
Digestive diseases	Digestive diseases	IBS	Gallbladder problems	17.380	2.196	1.614	2.936
Pulmonary diseases	Psychiatric conditions	Asthma	PTSD	19.122	2.180	1.539	3.023
Neurological diseases	Psychiatric conditions	Bothersome tinnitus	Burnout	4.273	2.172	1.595	2.913
Digestive diseases	Psychiatric conditions	IBS	Burnout	15.816	2.139	1.825	2.497
Musculoskeletal diseases	Musculoskeletal diseases	Chronic back pain	Osteoarthritis	14.106	2.138	1.649	2.747
Psychiatric conditions	Digestive diseases	Burnout	IBS	15.877	2.127	1.815	2.485
Digestive diseases	Musculoskeletal diseases	Gastritis	Chronic shoulder pain	28.451	2.126	1.787	2.520
Digestive diseases	Psychiatric conditions	IBS	GAD	15.678	2.121	1.809	2.479
Psychiatric conditions	Skin diseases	PTSD	Acne	2.658	2.117	1.545	2.862
Musculoskeletal diseases	Digestive diseases	Chronic shoulder pain	Gastritis	8.152	2.112	1.776	2.504
Psychiatric conditions	Digestive diseases	GAD	IBS	16.936	2.103	1.793	2.458
Psychiatric conditions	Digestive diseases	OCD	Gastric acid reflux	3.331	2.101	1.559	2.800
Digestive diseases	Psychiatric conditions	Gastric acid reflux	OCD	27.920	2.099	1.557	2.799
Musculoskeletal diseases	Psychiatric conditions	Sciatica	GAD	9.283	2.099	1.709	2.560
Neurological diseases	Psychiatric conditions	Dyslexia	Burnout	5.105	2.098	1.565	2.769
Psychiatric conditions	Musculoskeletal diseases	GAD	Sciatica	15.293	2.098	1.710	2.558
Psychiatric conditions	Neurological diseases	Burnout	Dyslexia	16.547	2.093	1.560	2.764
Digestive diseases	Cardiovascular diseases	Lactose intolerance	Cardiac arrhythmia	13.383	2.078	1.558	2.729
Cardiovascular diseases	Digestive diseases	Cardiac arrhythmia	Lactose intolerance	3.980	2.074	1.555	2.724
Skin diseases	Psychiatric conditions	Acne	PTSD	25.392	2.074	1.510	2.810
Digestive diseases	Cardiovascular diseases	Gastritis	Cardiac arrhythmia	27.881	2.061	1.644	2.568
Psychiatric conditions	Neurological diseases	Depression	Bothersome tinnitus	31.633	2.048	1.580	2.637
Digestive diseases	Musculoskeletal diseases	Gastritis	Chronic back pain	26.145	2.046	1.752	2.384
Digestive diseases	Psychiatric conditions	Gastric acid reflux	PTSD	27.586	2.045	1.506	2.742
Pulmonary diseases	Musculoskeletal diseases	Asthma	Chronic shoulder pain	17.808	2.039	1.654	2.495
Musculoskeletal diseases	Digestive diseases	Chronic back pain	Gastritis	9.481	2.035	1.742	2.371
Psychiatric conditions	Digestive diseases	PTSD	Gastric acid reflux	2.991	2.029	1.494	2.720
Cardiovascular diseases	Digestive diseases	Cardiac arrhythmia	Gastritis	4.528	2.028	1.618	2.525

Group of Condition 1	Group of Condition 2	Condition 1 …	… in individuals with Condition 2	Prevalence (%)	Odds ratio	Lower 95%-CI	Upper 95%-CI
Musculoskeletal diseases	Pulmonary diseases	Chronic shoulder pain	Asthma	8.350	2.027	1.644	2.481
Psychiatric conditions	Musculoskeletal diseases	Panic disorder	Chronic back pain	18.651	2.022	1.673	2.430
Psychiatric conditions	Digestive diseases	Depression	Stomach ulcer	37.544	2.018	1.486	2.717
Digestive diseases	Psychiatric conditions	Stomach ulcer	Depression	2.758	2.017	1.486	2.715
Psychiatric conditions	Musculoskeletal diseases	Panic disorder	Sciatica	16.568	2.011	1.656	2.429
Digestive diseases	Musculoskeletal diseases	IBS	Chronic back pain	15.987	2.009	1.660	2.417
Musculoskeletal diseases	Digestive diseases	Chronic back pain	IBS	10.696	2.006	1.657	2.413
Musculoskeletal diseases	Psychiatric conditions	Chronic back pain	Panic disorder	10.759	2.003	1.656	2.408
Neurological diseases	Psychiatric conditions	Bothersome tinnitus	Depression	3.196	1.997	1.541	2.571
Psychiatric conditions	Cardiovascular diseases	Depression	Cardiac arrhythmia	32.216	1.995	1.598	2.477
Musculoskeletal diseases	Psychiatric conditions	Sciatica	Panic disorder	9.366	1.990	1.637	2.405
Psychiatric conditions	Musculoskeletal diseases	Panic disorder	Chronic shoulder pain	19.705	1.988	1.605	2.443
Digestive diseases	Psychiatric conditions	Celiac disease	Depression	2.835	1.988	1.501	2.610
Psychiatric conditions	Cardiovascular diseases	Burnout	Cardiac arrhythmia	19.181	1.987	1.514	2.573
Neurological diseases	Cardiovascular diseases	Migraine	Cardiac arrhythmia	26.443	1.982	1.574	2.476
Psychiatric conditions	Digestive diseases	Depression	Celiac disease	31.609	1.977	1.491	2.598
Cardiovascular diseases	Psychiatric conditions	Cardiac arrhythmia	Depression	4.459	1.972	1.581	2.446
Musculoskeletal diseases	Psychiatric conditions	Chronic shoulder pain	Panic disorder	8.982	1.971	1.591	2.424
Cardiovascular diseases	Neurological diseases	Cardiac arrhythmia	Migraine	4.482	1.969	1.565	2.461
Musculoskeletal diseases	Musculoskeletal diseases	Chronic shoulder pain	Osteoarthritis	10.665	1.965	1.461	2.609
Digestive diseases	Psychiatric conditions	Gastric acid reflux	GAD	26.044	1.957	1.708	2.238
Psychiatric conditions	Digestive diseases	GAD	Gastric acid reflux	17.165	1.954	1.705	2.234
Psychiatric conditions	Musculoskeletal diseases	Depression	Sciatica	30.246	1.939	1.663	2.254
Cardiovascular diseases	Psychiatric conditions	Cardiac arrhythmia	Burnout	5.716	1.930	1.468	2.503
Psychiatric conditions	Musculoskeletal diseases	Burnout	Sciatica	17.247	1.922	1.596	2.301
Pulmonary diseases	Musculoskeletal diseases	Asthma	Chronic back pain	16.903	1.920	1.589	2.305
Pulmonary diseases	Digestive diseases	Asthma	Lactose intolerance	16.750	1.919	1.644	2.233
Digestive diseases	Pulmonary diseases	Lactose intolerance	Asthma	14.970	1.917	1.642	2.230
Digestive diseases	Cardiovascular diseases	Gastric acid reflux	Cardiac arrhythmia	24.721	1.907	1.502	2.402
Musculoskeletal diseases	Pulmonary diseases	Chronic back pain	Asthma	10.030	1.906	1.577	2.289
Digestive diseases	Musculoskeletal diseases	Gastric acid reflux	Chronic back pain	24.729	1.905	1.617	2.236
Digestive diseases	Musculoskeletal diseases	Gastritis	Sciatica	24.809	1.903	1.625	2.222
Digestive diseases	Psychiatric conditions	Gastric acid reflux	Social anxiety	26.987	1.898	1.508	2.372
Psychiatric conditions	Digestive diseases	Social anxiety	Gastric acid reflux	5.540	1.898	1.508	2.372
Digestive diseases	Musculoskeletal diseases	Gastric acid reflux	Chronic shoulder pain	26.765	1.898	1.578	2.272
Digestive diseases	Psychiatric conditions	IBS	Social anxiety	16.556	1.896	1.442	2.458
Cardiovascular diseases	Digestive diseases	Cardiac arrhythmia	Gastric acid reflux	4.518	1.894	1.491	2.386
Psychiatric conditions	Digestive diseases	Panic disorder	Lactose intolerance	17.644	1.890	1.614	2.206
Musculoskeletal diseases	Digestive diseases	Chronic back pain	Gastric acid reflux	10.095	1.888	1.602	2.217
Psychiatric conditions	Digestive diseases	Burnout	Lactose intolerance	13.993	1.885	1.588	2.227
Digestive diseases	Psychiatric conditions	Gastric acid reflux	Depression	23.119	1.885	1.699	2.090
Psychiatric conditions	Digestive diseases	Depression	Gastric acid reflux	30.489	1.881	1.696	2.086
Musculoskeletal diseases	Digestive diseases	Chronic shoulder pain	Gastric acid reflux	8.634	1.880	1.563	2.250
Psychiatric conditions	Digestive diseases	Social anxiety	IBS	5.571	1.879	1.429	2.437
Musculoskeletal diseases	Psychiatric conditions	Sciatica	Depression	9.175	1.878	1.611	2.183
Digestive diseases	Psychiatric conditions	Lactose intolerance	Panic disorder	15.322	1.876	1.602	2.190
Digestive diseases	Psychiatric conditions	Lactose intolerance	Burnout	14.040	1.868	1.574	2.206
Musculoskeletal diseases	Digestive diseases	Sciatica	Gastritis	8.816	1.868	1.595	2.180
Musculoskeletal diseases	Musculoskeletal diseases	Osteoarthritis	Chronic back pain	10.241	1.865	1.440	2.395
Musculoskeletal diseases	Psychiatric conditions	Sciatica	Burnout	11.265	1.864	1.547	2.234

Group of Condition 1	Group of Condition 2	Condition 1 …	… in individuals with Condition 2	Prevalence (%)	Odds ratio	Lower 95%-CI	Upper 95%-CI
Musculoskeletal diseases	Cardiovascular diseases	Chronic shoulder pain	Hypertension	9.572	1.856	1.416	2.401
Digestive diseases	Musculoskeletal diseases	Gastric acid reflux	Sciatica	23.619	1.845	1.565	2.167
Urogenital diseases	Skin diseases	Recurring UTI	Acne	15.087	1.845	1.619	2.098
Skin diseases	Urogenital diseases	Acne	Recurring UTI	23.386	1.840	1.615	2.093
Digestive diseases	Endocrine diseases	IBS	Thyroid disease	14.581	1.832	1.487	2.239
Psychiatric conditions	Digestive diseases	Depression	Lactose intolerance	29.978	1.822	1.604	2.066
Musculoskeletal diseases	Digestive diseases	Sciatica	Gastric acid reflux	9.449	1.822	1.545	2.140
Endocrine diseases	Digestive diseases	Thyroid disease	IBS	8.241	1.820	1.478	2.224
Psychiatric conditions	Neurological diseases	Panic disorder	Migraine	16.319	1.820	1.598	2.069
Musculoskeletal diseases	Musculoskeletal diseases	Sciatica	Osteoarthritis	15.826	1.817	1.426	2.300
Neurological diseases	Psychiatric conditions	Migraine	Panic disorder	24.832	1.814	1.593	2.063
Digestive diseases	Skin diseases	Gastric acid reflux	Acne	21.318	1.812	1.617	2.028
Digestive diseases	Psychiatric conditions	Lactose intolerance	Depression	13.969	1.811	1.594	2.054
Skin diseases	Digestive diseases	Acne	Gastric acid reflux	22.079	1.804	1.610	2.019
Digestive diseases	Psychiatric conditions	Gastric acid reflux	Burnout	22.697	1.782	1.551	2.042
Digestive diseases	Digestive diseases	Gastritis	Lactose intolerance	26.479	1.781	1.561	2.028
Digestive diseases	Digestive diseases	Gastric acid reflux	Lactose intolerance	22.222	1.777	1.546	2.036
Digestive diseases	Neurological diseases	Gastritis	Migraine	24.179	1.775	1.598	1.970
Neurological diseases	Digestive diseases	Migraine	Gastritis	23.128	1.774	1.597	1.969
Digestive diseases	Digestive diseases	Lactose intolerance	Gastric acid reflux	13.655	1.772	1.543	2.031
Psychiatric conditions	Digestive diseases	Burnout	Gastric acid reflux	13.902	1.772	1.542	2.031
Digestive diseases	Digestive diseases	Lactose intolerance	Gastritis	14.458	1.772	1.553	2.018
Psychiatric conditions	Digestive diseases	Social anxiety	Gastritis	4.891	1.767	1.413	2.195
Musculoskeletal diseases	Digestive diseases	Chronic shoulder pain	Lactose intolerance	7.688	1.766	1.398	2.208
Digestive diseases	Psychiatric conditions	Gastritis	Social anxiety	26.821	1.766	1.413	2.194
Digestive diseases	Musculoskeletal diseases	Lactose intolerance	Chronic shoulder pain	14.647	1.763	1.395	2.204
Endocrine diseases	Digestive diseases	Thyroid disease	Lactose intolerance	6.689	1.757	1.400	2.182
Digestive diseases	Endocrine diseases	Lactose intolerance	Thyroid disease	11.921	1.754	1.399	2.179
Digestive diseases	Musculoskeletal diseases	IBS	Sciatica	14.019	1.751	1.435	2.122
Neurological diseases	Musculoskeletal diseases	Migraine	Sciatica	24.554	1.742	1.480	2.043
Urogenital diseases	Digestive diseases	Recurring UTI	Gastric acid reflux	15.149	1.741	1.523	1.986
Musculoskeletal diseases	Digestive diseases	Sciatica	IBS	9.192	1.737	1.423	2.104
Digestive diseases	Urogenital diseases	Gastric acid reflux	Recurring UTI	22.674	1.737	1.519	1.981
Digestive diseases	Skin diseases	Gastritis	Acne	23.385	1.719	1.543	1.914
Musculoskeletal diseases	Neurological diseases	Sciatica	Migraine	9.122	1.718	1.460	2.015
Psychiatric conditions	Musculoskeletal diseases	Depression	Osteoarthritis	22.477	1.707	1.387	2.093
Skin diseases	Digestive diseases	Acne	Gastritis	21.521	1.702	1.527	1.895
Digestive diseases	Skin diseases	Gastric acid reflux	Eczema	21.119	1.701	1.528	1.891
Psychiatric conditions	Skin diseases	Social anxiety	Acne	4.989	1.699	1.340	2.136
Neurological diseases	Psychiatric conditions	Migraine	Burnout	23.196	1.699	1.482	1.944
Digestive diseases	Pulmonary diseases	Gastritis	Asthma	23.666	1.699	1.498	1.924
Pulmonary diseases	Digestive diseases	Asthma	Gastritis	14.458	1.699	1.498	1.923
Skin diseases	Digestive diseases	Eczema	Gastric acid reflux	25.645	1.699	1.527	1.889
Psychiatric conditions	Neurological diseases	Burnout	Migraine	13.194	1.695	1.478	1.939
Digestive diseases	Neurological diseases	Gastric acid reflux	Migraine	21.591	1.695	1.516	1.894
Neurological diseases	Digestive diseases	Migraine	Gastric acid reflux	23.249	1.695	1.516	1.893
Psychiatric conditions	Digestive diseases	GAD	Lactose intolerance	16.095	1.680	1.422	1.977
Digestive diseases	Skin diseases	Gastric acid reflux	Herpes	20.641	1.677	1.512	1.860
Skin diseases	Digestive diseases	Herpes	Gastric acid reflux	26.698	1.676	1.511	1.859
Digestive diseases	Psychiatric conditions	Lactose intolerance	GAD	15.008	1.667	1.411	1.962

Group of Condition 1	Group of Condition 2	Condition 1 …	… in individuals with Condition 2	Prevalence (%)	Odds ratio	Lower 95%-CI	Upper 95%-CI
Neurological diseases	Musculoskeletal diseases	Migraine	Chronic back pain	23.897	1.665	1.412	1.956
Musculoskeletal diseases	Neurological diseases	Chronic back pain	Migraine	9.059	1.660	1.408	1.950
Pulmonary diseases	Musculoskeletal diseases	Asthma	Sciatica	13.254	1.657	1.359	2.007
Digestive diseases	Musculoskeletal diseases	Lactose intolerance	Sciatica	11.555	1.657	1.335	2.040
Musculoskeletal diseases	Digestive diseases	Sciatica	Lactose intolerance	7.522	1.657	1.335	2.040
Musculoskeletal diseases	Digestive diseases	Chronic back pain	Lactose intolerance	9.292	1.656	1.341	2.027
Digestive diseases	Musculoskeletal diseases	Lactose intolerance	Chronic back pain	13.988	1.653	1.339	2.024
Musculoskeletal diseases	Pulmonary diseases	Sciatica	Asthma	7.708	1.649	1.352	1.997
Digestive diseases	Skin diseases	Gastritis	Eczema	22.993	1.638	1.479	1.813
Skin diseases	Digestive diseases	Eczema	Gastritis	24.811	1.637	1.478	1.811
Psychiatric conditions	Neurological diseases	Depression	Migraine	27.210	1.624	1.464	1.800
Neurological diseases	Psychiatric conditions	Migraine	Depression	22.216	1.623	1.463	1.798
Pulmonary diseases	Psychiatric conditions	Asthma	Depression	14.021	1.615	1.428	1.824
Psychiatric conditions	Pulmonary diseases	Depression	Asthma	26.877	1.614	1.427	1.822
Neurological diseases	Digestive diseases	Migraine	Lactose intolerance	22.124	1.606	1.399	1.839
Digestive diseases	Neurological diseases	Lactose intolerance	Migraine	12.626	1.602	1.395	1.834
Psychiatric conditions	Skin diseases	Depression	Acne	26.715	1.597	1.436	1.775
Digestive diseases	Cardiovascular diseases	Gastric acid reflux	Hypertension	20.956	1.588	1.314	1.910
Skin diseases	Psychiatric conditions	Acne	Depression	20.979	1.577	1.418	1.752
Cardiovascular diseases	Digestive diseases	Hypertension	Gastric acid reflux	6.997	1.558	1.290	1.872
Skin diseases	Skin diseases	Herpes	Acne	24.697	1.544	1.389	1.713
Neurological diseases	Cardiovascular diseases	Migraine	Hypertension	22.301	1.538	1.275	1.845
Urogenital diseases	Psychiatric conditions	Recurring UTI	Panic disorder	15.178	1.531	1.305	1.789
Skin diseases	Skin diseases	Acne	Herpes	19.774	1.527	1.375	1.695
Skin diseases	Urogenital diseases	Herpes	Recurring UTI	26.792	1.522	1.344	1.721
Urogenital diseases	Skin diseases	Recurring UTI	Herpes	13.839	1.520	1.343	1.718
Psychiatric conditions	Urogenital diseases	Panic disorder	Recurring UTI	16.073	1.518	1.293	1.774
Skin diseases	Skin diseases	Eczema	Acne	24.434	1.507	1.354	1.675
Skin diseases	Skin diseases	Acne	Eczema	20.839	1.505	1.352	1.673
Skin diseases	Urogenital diseases	Eczema	Recurring UTI	24.555	1.501	1.321	1.703
Urogenital diseases	Skin diseases	Recurring UTI	Eczema	13.510	1.501	1.321	1.703
Digestive diseases	Skin diseases	IBS	Acne	12.037	1.496	1.299	1.718
Skin diseases	Digestive diseases	Acne	IBS	20.434	1.493	1.296	1.714
Digestive diseases	Pulmonary diseases	Gastric acid reflux	Asthma	19.812	1.493	1.304	1.706
Pulmonary diseases	Digestive diseases	Asthma	Gastric acid reflux	13.621	1.492	1.302	1.704
Digestive diseases	Skin diseases	IBS	Eczema	12.000	1.486	1.303	1.692
Skin diseases	Digestive diseases	Eczema	IBS	23.886	1.486	1.302	1.692
Skin diseases	Skin diseases	Herpes	Eczema	24.895	1.486	1.347	1.638
Skin diseases	Digestive diseases	Herpes	Gastritis	25.174	1.485	1.343	1.640
Digestive diseases	Skin diseases	Gastritis	Herpes	21.901	1.485	1.343	1.640
Skin diseases	Skin diseases	Eczema	Herpes	23.372	1.484	1.345	1.636
Digestive diseases	Pulmonary diseases	IBS	Asthma	12.204	1.480	1.255	1.738
Pulmonary diseases	Digestive diseases	Asthma	IBS	13.753	1.476	1.252	1.733
Skin diseases	Psychiatric conditions	Acne	GAD	22.073	1.475	1.281	1.694
Psychiatric conditions	Skin diseases	GAD	Acne	14.047	1.474	1.280	1.694
Digestive diseases	Urogenital diseases	IBS	Recurring UTI	12.862	1.466	1.242	1.721
Skin diseases	Psychiatric conditions	Eczema	GAD	24.652	1.466	1.282	1.673
Urogenital diseases	Digestive diseases	Recurring UTI	IBS	14.087	1.465	1.242	1.721
Psychiatric conditions	Skin diseases	Burnout	Acne	10.436	1.463	1.257	1.698
Psychiatric conditions	Skin diseases	GAD	Eczema	13.374	1.462	1.279	1.668

Group of Condition 1	Group of Condition 2	Condition 1 …	… in individuals with Condition 2	Prevalence (%)	Odds ratio	Lower 95%-CI	Upper 95%-CI
Pulmonary diseases	Neurological diseases	Asthma	Migraine	13.100	1.445	1.265	1.647
Neurological diseases	Pulmonary diseases	Migraine	Asthma	20.504	1.444	1.264	1.646
Skin diseases	Digestive diseases	Eczema	Lactose intolerance	23.494	1.438	1.258	1.641
Digestive diseases	Skin diseases	Lactose intolerance	Eczema	11.888	1.438	1.257	1.640
Urogenital diseases	Psychiatric conditions	Recurring UTI	Depression	13.454	1.437	1.266	1.629
Skin diseases	Psychiatric conditions	Acne	Burnout	17.647	1.431	1.229	1.660
Psychiatric conditions	Neurological diseases	GAD	Migraine	13.352	1.428	1.239	1.641
Psychiatric conditions	Urogenital diseases	Depression	Recurring UTI	26.551	1.427	1.256	1.617
Neurological diseases	Psychiatric conditions	Migraine	GAD	21.815	1.426	1.236	1.639
Psychiatric conditions	Skin diseases	Depression	Eczema	25.042	1.418	1.282	1.567
Skin diseases	Psychiatric conditions	Eczema	Depression	23.067	1.416	1.281	1.565
Skin diseases	Psychiatric conditions	Herpes	GAD	24.446	1.392	1.218	1.587
Psychiatric conditions	Skin diseases	GAD	Herpes	12.447	1.383	1.210	1.576

Abbreviations: CI = Confidence Interval; GAD = Generalized Anxiety Disorder; IBS = Irritable Bowel Syndrome; OCD = Obsessive-Compulsive Disorder; PTSD = Posttraumatic Stress Disorder; UTI = Urinary Tract Infection.
