# Supplementary material for: Sex Differences in Comorbidity Combinations in the Swedish Population
Source: Biomolecules. 2022 Jul 6;12(7):949. doi: 10.3390/biom12070949 (PMC9313065; doi:10.3390/biom12070949)
Supplement: Supplementary file 1 [file biomolecules-12-00949-s001.zip › Table S2.pdf]

Table S2. Significantly elevated comorbidity combinations ($p<0.05/5724$) in male participants (N=11960) sorted by odds ratio.

Group of Condition 1	Group of Condition 2	Condition 1 …	… in individuals with Condition 2	Prevalence (%)	Odds ratio	Lower 95%-CI	Upper 95%-CI
Psychiatric conditions	Psychiatric conditions	Autism	Asperger’s syndrome	12.245	239.053	20.329	2127.416
Psychiatric conditions	Psychiatric conditions	Asperger’s syndrome	Autism	54.545	224.575	22.004	2157.836
Psychiatric conditions	Psychiatric conditions	Panic disorder	Agoraphobia	80.645	66.537	26.607	202.097
Psychiatric conditions	Psychiatric conditions	Agoraphobia	Panic disorder	3.516	65.792	26.376	199.464
Psychiatric conditions	Psychiatric conditions	Social anxiety	Agoraphobia	51.613	46.104	20.464	104.423
Psychiatric conditions	Psychiatric conditions	Agoraphobia	Social anxiety	5.424	45.427	20.501	101.309
Psychiatric conditions	Psychiatric conditions	Agoraphobia	OCD	6.154	26.520	9.298	65.686
Psychiatric conditions	Psychiatric conditions	OCD	Agoraphobia	25.806	25.077	8.603	63.945
Psychiatric conditions	Psychiatric conditions	Social anxiety	Depression	15.867	23.560	17.434	32.232
Psychiatric conditions	Psychiatric conditions	Depression	Social anxiety	72.881	23.485	17.382	32.119
Psychiatric conditions	Psychiatric conditions	Depression	Bipolar disease	73.469	19.855	9.643	44.163
Psychiatric conditions	Psychiatric conditions	Bipolar disease	Depression	2.657	19.390	9.532	42.676
Psychiatric conditions	Psychiatric conditions	GAD	OCD	50.000	18.732	12.243	28.716
Psychiatric conditions	Psychiatric conditions	OCD	GAD	9.774	18.597	12.176	28.469
Psychiatric conditions	Psychiatric conditions	Depression	GAD	64.662	18.508	15.230	22.549
Psychiatric conditions	Psychiatric conditions	GAD	Depression	31.734	18.493	15.218	22.531
Psychiatric conditions	Psychiatric conditions	GAD	Panic disorder	41.210	17.327	14.052	21.361
Psychiatric conditions	Psychiatric conditions	Panic disorder	GAD	44.060	17.315	14.043	21.346
Neurological diseases	Neurological diseases	Meniere’s disease	Bothersome tinnitus	1.656	17.218	4.703	56.835
Neurological diseases	Neurological diseases	Bothersome tinnitus	Meniere’s disease	33.333	16.954	4.644	56.003
Psychiatric conditions	Psychiatric conditions	Bipolar disease	OCD	5.385	16.419	6.157	38.674
Psychiatric conditions	Psychiatric conditions	Social anxiety	GAD	21.353	16.225	12.169	21.605
Psychiatric conditions	Psychiatric conditions	GAD	Social anxiety	48.136	16.224	12.165	21.609
Psychiatric conditions	Psychiatric conditions	OCD	Bipolar disease	14.286	15.660	5.781	37.400
Psychiatric conditions	Psychiatric conditions	Panic disorder	OCD	45.385	15.508	10.100	23.777
Psychiatric conditions	Psychiatric conditions	OCD	Panic disorder	8.298	15.342	10.014	23.465
Psychiatric conditions	Psychiatric conditions	Social anxiety	OCD	26.923	14.412	8.717	23.227
Psychiatric conditions	Psychiatric conditions	OCD	Social anxiety	11.864	14.363	8.697	23.126
Psychiatric conditions	Psychiatric conditions	Social anxiety	Panic disorder	18.987	14.167	10.611	18.879
Psychiatric conditions	Psychiatric conditions	Panic disorder	Social anxiety	45.763	14.152	10.599	18.860
Psychiatric conditions	Psychiatric conditions	Depression	Burnout	56.613	13.867	11.382	16.919
Psychiatric conditions	Psychiatric conditions	Burnout	Depression	25.904	13.625	11.191	16.610
Musculoskeletal diseases	Musculoskeletal diseases	Chronic back pain	Chronic shoulder pain	41.155	13.572	10.039	18.267
Musculoskeletal diseases	Musculoskeletal diseases	Chronic shoulder pain	Chronic back pain	19.689	13.509	9.991	18.184
Psychiatric conditions	Psychiatric conditions	Bipolar disease	Social anxiety	4.746	12.605	5.708	26.121
Psychiatric conditions	Psychiatric conditions	Bipolar disease	Burnout	3.065	12.432	6.233	24.293
Psychiatric conditions	Psychiatric conditions	Social anxiety	Bipolar disease	28.571	12.230	5.459	25.674
Psychiatric conditions	Psychiatric conditions	PTSD	Panic disorder	5.063	12.162	6.826	21.538
Psychiatric conditions	Psychiatric conditions	Panic disorder	PTSD	50.704	12.091	6.733	21.599
Psychiatric conditions	Psychiatric conditions	Agoraphobia	GAD	2.406	11.978	5.383	26.096
Psychiatric conditions	Psychiatric conditions	GAD	Agoraphobia	51.613	11.954	5.318	26.310
Psychiatric conditions	Psychiatric conditions	GAD	PTSD	45.070	11.953	6.629	21.357
Psychiatric conditions	Psychiatric conditions	Depression	Agoraphobia	64.516	11.866	5.393	27.413
Psychiatric conditions	Psychiatric conditions	Agoraphobia	Depression	1.476	11.845	5.428	27.202
Cardiovascular diseases	Cardiovascular diseases	Hyperlipidemia	Angina pectoris	44.828	11.835	4.799	28.981
Psychiatric conditions	Psychiatric conditions	Asperger’s syndrome	Social anxiety	4.407	11.779	4.991	26.157
Psychiatric conditions	Psychiatric conditions	Social anxiety	Asperger’s syndrome	26.531	11.774	4.889	26.543
Psychiatric conditions	Psychiatric conditions	Burnout	Bipolar disease	38.776	11.637	5.784	22.903

Group of Condition 1	Group of Condition 2	Condition 1 …	… in individuals with Condition 2	Prevalence (%)	Odds ratio	Lower 95%-CI	Upper 95%-CI
Psychiatric conditions	Psychiatric conditions	PTSD	GAD	4.812	11.522	6.442	20.395
Cardiovascular diseases	Cardiovascular diseases	Angina pectoris	Hyperlipidemia	3.325	11.433	4.473	28.953
Psychiatric conditions	Psychiatric conditions	OCD	Depression	5.461	11.330	7.449	17.421
Psychiatric conditions	Psychiatric conditions	Depression	OCD	56.923	11.316	7.420	17.435
Psychiatric conditions	Psychiatric conditions	Depression	Panic disorder	55.134	11.209	9.309	13.505
Psychiatric conditions	Psychiatric conditions	Panic disorder	Depression	28.930	11.194	9.298	13.486
Digestive diseases	Digestive diseases	Fecal incontinence	Stomach ulcer	4.110	11.119	4.093	25.462
Digestive diseases	Digestive diseases	Stomach ulcer	Fecal incontinence	9.836	11.006	4.051	25.216
Psychiatric conditions	Psychiatric conditions	Depression	PTSD	66.197	10.255	5.783	18.508
Psychiatric conditions	Psychiatric conditions	Bipolar disease	GAD	2.857	10.216	5.115	19.974
Psychiatric conditions	Psychiatric conditions	GAD	Bipolar disease	38.776	10.137	4.997	20.133
Psychiatric conditions	Psychiatric conditions	PTSD	Depression	3.469	10.112	5.735	18.157
Digestive diseases	Digestive diseases	Lactose intolerance	Celiac disease	33.684	9.963	6.044	16.056
Digestive diseases	Digestive diseases	Celiac disease	Lactose intolerance	4.938	9.947	6.035	16.026
Psychiatric conditions	Psychiatric conditions	Bipolar disease	Panic disorder	2.532	9.670	4.827	18.983
Psychiatric conditions	Psychiatric conditions	Panic disorder	Bipolar disease	36.735	9.479	4.679	18.778
Psychiatric conditions	Psychiatric conditions	OCD	PTSD	11.268	9.460	3.404	22.302
Cardiovascular diseases	Cardiovascular diseases	Hypertension	Angina pectoris	44.828	9.132	3.623	23.001
Digestive diseases	Digestive diseases	Gastritis	Stomach ulcer	41.781	9.095	6.273	13.109
Digestive diseases	Digestive diseases	Stomach ulcer	Gastritis	6.846	9.062	6.248	13.065
Psychiatric conditions	Psychiatric conditions	Panic disorder	Burnout	31.290	8.818	7.045	11.007
Psychiatric conditions	Psychiatric conditions	GAD	Burnout	28.710	8.757	6.957	10.987
Psychiatric conditions	Psychiatric conditions	Agoraphobia	Burnout	1.452	8.754	3.661	19.541
Psychiatric conditions	Psychiatric conditions	Burnout	Panic disorder	27.286	8.696	6.943	10.859
Psychiatric conditions	Psychiatric conditions	Burnout	Agoraphobia	29.032	8.689	3.608	19.566
Psychiatric conditions	Psychiatric conditions	Burnout	GAD	26.767	8.668	6.879	10.887
Cardiovascular diseases	Cardiovascular diseases	Angina pectoris	Hypertension	2.170	8.612	3.341	22.389
Psychiatric conditions	Musculoskeletal diseases	PTSD	Chronic shoulder pain	5.415	7.967	3.483	16.410
Psychiatric conditions	Psychiatric conditions	Social anxiety	PTSD	21.127	7.944	3.664	15.900
Psychiatric conditions	Psychiatric conditions	PTSD	Social anxiety	5.085	7.940	3.677	15.830
Psychiatric conditions	Psychiatric conditions	Social anxiety	Burnout	13.065	7.713	5.589	10.536
Psychiatric conditions	Psychiatric conditions	Burnout	Social anxiety	27.458	7.614	5.506	10.427
Musculoskeletal diseases	Psychiatric conditions	Chronic shoulder pain	PTSD	21.127	7.487	3.274	15.457
Psychiatric conditions	Psychiatric conditions	Burnout	OCD	23.846	6.827	4.109	11.018
Psychiatric conditions	Psychiatric conditions	OCD	Burnout	5.000	6.757	4.106	10.783
Cardiovascular diseases	Cardiovascular diseases	Hyperlipidemia	Hypertension	23.205	6.366	4.852	8.316
Psychiatric conditions	Urogenital diseases	OCD	Prostate problems	3.216	6.314	2.935	12.302
Psychiatric conditions	Psychiatric conditions	Asperger’s syndrome	Depression	1.919	6.247	2.947	13.357
Psychiatric conditions	Neurological diseases	OCD	Dyslexia	4.585	6.220	3.361	10.794
Neurological diseases	Psychiatric conditions	Dyslexia	OCD	12.308	6.219	3.364	10.778
Urogenital diseases	Cardiovascular diseases	Recurring UTI	Hypertension	2.003	6.211	2.665	13.220
Cardiovascular diseases	Cardiovascular diseases	Hypertension	Hyperlipidemia	35.550	6.127	4.642	8.052
Psychiatric conditions	Psychiatric conditions	PTSD	Burnout	3.387	6.110	3.195	11.183
Psychiatric conditions	Psychiatric conditions	Depression	Asperger’s syndrome	53.061	5.833	2.680	12.697
Psychiatric conditions	Psychiatric conditions	Burnout	PTSD	29.577	5.805	3.033	10.634
Cardiovascular diseases	Endocrine diseases	Hyperlipidemia	Diabetes	24.675	5.751	3.004	10.537
Endocrine diseases	Cardiovascular diseases	Diabetes	Hyperlipidemia	4.859	5.720	2.939	10.636
Urogenital diseases	Psychiatric conditions	Prostate problems	OCD	8.462	5.546	2.546	10.952
Psychiatric conditions	Digestive diseases	Burnout	Fecal incontinence	21.311	5.499	2.574	10.923
Psychiatric conditions	Urogenital diseases	GAD	Recurring UTI	21.569	5.477	2.559	10.887

Group of Condition 1	Group of Condition 2	Condition 1 …	… in individuals with Condition 2	Prevalence (%)	Odds ratio	Lower 95%-CI	Upper 95%-CI
Digestive diseases	Psychiatric conditions	Fecal incontinence	Burnout	2.097	5.451	2.578	10.672
Urogenital diseases	Psychiatric conditions	Recurring UTI	GAD	1.654	5.384	2.496	10.793
Musculoskeletal diseases	Musculoskeletal diseases	Chronic back pain	Sciatica	21.348	5.253	4.028	6.796
Musculoskeletal diseases	Musculoskeletal diseases	Sciatica	Chronic back pain	19.689	5.137	3.937	6.650
Digestive diseases	Digestive diseases	Gastritis	Gastric acid reflux	21.529	4.764	4.041	5.609
Digestive diseases	Digestive diseases	Gastric acid reflux	Gastritis	37.935	4.761	4.038	5.606
Urogenital diseases	Digestive diseases	Recurring UTI	Gastritis	1.347	4.658	2.282	8.905
Digestive diseases	Digestive diseases	Celiac disease	IBS	2.771	4.643	2.295	8.549
Digestive diseases	Digestive diseases	IBS	Celiac disease	12.632	4.581	2.263	8.441
Skin diseases	Digestive diseases	Eczema	Fecal incontinence	32.787	4.523	2.464	8.139
Digestive diseases	Skin diseases	Fecal incontinence	Eczema	1.254	4.489	2.446	8.077
Endocrine diseases	Cardiovascular diseases	Thyroid disease	Hyperlipidemia	3.581	4.454	2.231	8.324
Psychiatric conditions	Musculoskeletal diseases	GAD	Chronic shoulder pain	17.690	4.272	2.925	6.105
Digestive diseases	Digestive diseases	Stomach ulcer	Gastric acid reflux	3.567	4.243	2.922	6.110
Digestive diseases	Digestive diseases	Gastric acid reflux	Stomach ulcer	38.356	4.236	2.918	6.100
Urogenital diseases	Skin diseases	Recurring UTI	Acne	1.160	4.164	2.187	7.724
Digestive diseases	Cardiovascular diseases	Stomach ulcer	Cardiac arrhythmia	4.762	4.160	2.184	7.307
Digestive diseases	Digestive diseases	Gastritis	Gallbladder problems	23.232	4.136	2.443	6.735
Cardiovascular diseases	Digestive diseases	Cardiac arrhythmia	Stomach ulcer	9.589	4.083	2.144	7.172
Musculoskeletal diseases	Psychiatric conditions	Chronic shoulder pain	GAD	7.368	4.082	2.793	5.840
Neurological diseases	Psychiatric conditions	Dyslexia	Social anxiety	10.508	4.041	2.515	6.228
Digestive diseases	Digestive diseases	Gallbladder problems	Gastritis	2.581	4.022	2.375	6.549
Psychiatric conditions	Neurological diseases	Social anxiety	Dyslexia	8.883	3.992	2.482	6.159
Psychiatric conditions	Musculoskeletal diseases	Burnout	Chronic shoulder pain	20.578	3.760	2.590	5.341
Neurological diseases	Psychiatric conditions	Dyslexia	Burnout	8.871	3.731	2.621	5.205
Psychiatric conditions	Neurological diseases	Burnout	Dyslexia	15.759	3.730	2.618	5.208
Musculoskeletal diseases	Psychiatric conditions	Chronic shoulder pain	Burnout	9.194	3.687	2.537	5.245
Digestive diseases	Psychiatric conditions	IBS	Panic disorder	9.986	3.553	2.619	4.751
Digestive diseases	Digestive diseases	Lactose intolerance	Stomach ulcer	14.384	3.508	2.087	5.613
Digestive diseases	Digestive diseases	Stomach ulcer	Lactose intolerance	3.241	3.504	2.086	5.604
Psychiatric conditions	Digestive diseases	Panic disorder	IBS	16.397	3.500	2.578	4.683
Digestive diseases	Psychiatric conditions	IBS	Social anxiety	9.831	3.459	2.186	5.255
Psychiatric conditions	Musculoskeletal diseases	Depression	Chronic shoulder pain	31.408	3.393	2.470	4.609
Psychiatric conditions	Digestive diseases	Social anxiety	IBS	6.697	3.379	2.135	5.136
Musculoskeletal diseases	Musculoskeletal diseases	Chronic shoulder pain	Osteoarthritis	9.000	3.350	2.110	5.148
Cardiovascular diseases	Cardiovascular diseases	Cardiac arrhythmia	Hypertension	8.013	3.331	2.279	4.777
Cardiovascular diseases	Cardiovascular diseases	Hypertension	Cardiac arrhythmia	16.327	3.306	2.259	4.750
Musculoskeletal diseases	Psychiatric conditions	Chronic shoulder pain	Depression	6.421	3.293	2.394	4.478
Psychiatric conditions	Musculoskeletal diseases	Social anxiety	Chronic back pain	6.390	3.270	2.139	4.837
Musculoskeletal diseases	Psychiatric conditions	Chronic back pain	Social anxiety	12.542	3.241	2.119	4.800
Musculoskeletal diseases	Neurological diseases	Chronic shoulder pain	Bothersome tinnitus	8.278	3.197	1.894	5.127
Digestive diseases	Digestive diseases	Gastric acid reflux	Gallbladder problems	30.303	3.158	1.954	4.983
Digestive diseases	Psychiatric conditions	Gastritis	Burnout	18.871	3.149	2.478	3.969
Digestive diseases	Digestive diseases	Gallbladder problems	Gastric acid reflux	1.911	3.126	1.934	4.930
Psychiatric conditions	Digestive diseases	Burnout	Gastritis	13.131	3.124	2.459	3.938
Musculoskeletal diseases	Musculoskeletal diseases	Osteoarthritis	Chronic shoulder pain	12.996	3.007	1.891	4.636
Digestive diseases	Psychiatric conditions	IBS	GAD	8.271	2.967	2.131	4.051
Psychiatric conditions	Cardiovascular diseases	Burnout	Cardiac arrhythmia	13.605	2.931	1.999	4.188
Psychiatric conditions	Digestive diseases	GAD	IBS	12.702	2.927	2.102	3.999
Psychiatric conditions	Cardiovascular diseases	Social anxiety	Hypertension	4.674	2.916	1.792	4.557

Group of Condition 1	Group of Condition 2	Condition 1 …	… in individuals with Condition 2	Prevalence (%)	Odds ratio	Lower 95%-CI	Upper 95%-CI
Neurological diseases	Psychiatric conditions	Dyslexia	GAD	7.368	2.915	2.008	4.129
Psychiatric conditions	Neurological diseases	GAD	Dyslexia	14.040	2.894	1.992	4.104
Psychiatric conditions	Neurological diseases	GAD	Bothersome tinnitus	13.576	2.857	1.900	4.174
Cardiovascular diseases	Psychiatric conditions	Cardiac arrhythmia	Burnout	6.452	2.850	1.940	4.079
Psychiatric conditions	Urogenital diseases	GAD	Prostate problems	9.942	2.811	1.824	4.192
Psychiatric conditions	Musculoskeletal diseases	GAD	Chronic back pain	12.781	2.804	2.068	3.743
Musculoskeletal diseases	Psychiatric conditions	Chronic back pain	GAD	11.128	2.768	2.041	3.697
Digestive diseases	Digestive diseases	IBS	Lactose intolerance	8.179	2.764	1.967	3.799
Digestive diseases	Cardiovascular diseases	Gastritis	Cardiac arrhythmia	18.027	2.754	1.952	3.809
Musculoskeletal diseases	Musculoskeletal diseases	Chronic shoulder pain	Sciatica	7.865	2.752	1.797	4.081
Neurological diseases	Psychiatric conditions	Dyslexia	Depression	6.494	2.748	2.047	3.648
Digestive diseases	Digestive diseases	Lactose intolerance	IBS	12.240	2.746	1.954	3.773
Psychiatric conditions	Neurological diseases	Depression	Dyslexia	25.215	2.734	2.034	3.634
Digestive diseases	Psychiatric conditions	IBS	Depression	7.159	2.710	2.088	3.485
Digestive diseases	Digestive diseases	IBS	Gastritis	8.642	2.709	2.021	3.578
Digestive diseases	Digestive diseases	Gastritis	IBS	17.783	2.705	2.018	3.573
Musculoskeletal diseases	Neurological diseases	Chronic shoulder pain	Migraine	5.282	2.699	1.820	3.890
Cardiovascular diseases	Digestive diseases	Cardiac arrhythmia	Gastritis	5.948	2.691	1.906	3.723
Neurological diseases	Musculoskeletal diseases	Migraine	Chronic shoulder pain	16.245	2.689	1.812	3.876
Psychiatric conditions	Digestive diseases	Depression	IBS	22.402	2.688	2.070	3.459
Neurological diseases	Psychiatric conditions	Bothersome tinnitus	GAD	6.165	2.682	1.782	3.921
Musculoskeletal diseases	Musculoskeletal diseases	Sciatica	Chronic shoulder pain	15.162	2.673	1.742	3.971
Psychiatric conditions	Musculoskeletal diseases	Burnout	Chronic back pain	13.472	2.631	1.953	3.492
Digestive diseases	Musculoskeletal diseases	Gastritis	Chronic shoulder pain	17.690	2.625	1.816	3.710
Musculoskeletal diseases	Psychiatric conditions	Chronic back pain	Burnout	12.581	2.616	1.941	3.472
Musculoskeletal diseases	Digestive diseases	Chronic shoulder pain	Gastritis	5.499	2.594	1.793	3.665
Psychiatric conditions	Digestive diseases	Panic disorder	Gastritis	13.917	2.579	2.023	3.258
Digestive diseases	Psychiatric conditions	Gastritis	Panic disorder	17.440	2.573	2.017	3.252
Psychiatric conditions	Digestive diseases	Panic disorder	Gastric acid reflux	11.975	2.558	2.091	3.116
Pulmonary diseases	Skin diseases	Asthma	Eczema	17.116	2.543	2.152	2.996
Digestive diseases	Psychiatric conditions	Gastric acid reflux	Panic disorder	26.442	2.542	2.077	3.097
Skin diseases	Pulmonary diseases	Eczema	Asthma	26.025	2.542	2.151	2.995
Digestive diseases	Musculoskeletal diseases	Gastritis	Chronic back pain	16.062	2.528	1.941	3.255
Psychiatric conditions	Musculoskeletal diseases	Depression	Chronic back pain	23.316	2.523	1.986	3.182
Psychiatric conditions	Cardiovascular diseases	Panic disorder	Cardiac arrhythmia	13.265	2.520	1.686	3.656
Musculoskeletal diseases	Digestive diseases	Chronic back pain	Gastritis	10.438	2.509	1.927	3.232
Psychiatric conditions	Musculoskeletal diseases	Panic disorder	Chronic back pain	13.644	2.504	1.849	3.339
Musculoskeletal diseases	Psychiatric conditions	Chronic back pain	Depression	9.963	2.500	1.967	3.152
Psychiatric conditions	Digestive diseases	GAD	Gastric acid reflux	11.274	2.482	2.016	3.042
Digestive diseases	Musculoskeletal diseases	Gastric acid reflux	Chronic shoulder pain	28.881	2.473	1.808	3.341
Musculoskeletal diseases	Psychiatric conditions	Chronic back pain	Panic disorder	11.111	2.467	1.821	3.291
Digestive diseases	Psychiatric conditions	Gastric acid reflux	GAD	26.617	2.467	2.003	3.025
Musculoskeletal diseases	Digestive diseases	Chronic shoulder pain	Gastric acid reflux	5.096	2.461	1.800	3.325
Neurological diseases	Neurological diseases	Migraine	Dyslexia	16.046	2.440	1.700	3.416
Neurological diseases	Neurological diseases	Dyslexia	Migraine	6.573	2.436	1.698	3.411
Psychiatric conditions	Neurological diseases	Burnout	Migraine	10.798	2.422	1.843	3.142
Cardiovascular diseases	Psychiatric conditions	Cardiac arrhythmia	Panic disorder	5.485	2.413	1.612	3.506
Neurological diseases	Psychiatric conditions	Migraine	Burnout	14.839	2.409	1.834	3.126
Psychiatric conditions	Musculoskeletal diseases	GAD	Sciatica	10.112	2.393	1.710	3.280
Psychiatric conditions	Digestive diseases	Depression	Gastritis	21.998	2.372	1.953	2.868

Group of Condition 1	Group of Condition 2	Condition 1 …	… in individuals with Condition 2	Prevalence (%)	Odds ratio	Lower 95%-CI	Upper 95%-CI
Digestive diseases	Psychiatric conditions	Gastritis	Depression	14.465	2.367	1.949	2.862
Neurological diseases	Psychiatric conditions	Migraine	Depression	12.989	2.354	1.925	2.864
Psychiatric conditions	Neurological diseases	Depression	Migraine	20.657	2.352	1.923	2.862
Digestive diseases	Digestive diseases	IBS	Gastric acid reflux	6.752	2.315	1.796	2.959
Digestive diseases	Digestive diseases	Gastric acid reflux	IBS	24.480	2.307	1.789	2.949
Digestive diseases	Urogenital diseases	Gastric acid reflux	Prostate problems	24.269	2.303	1.717	3.057
Psychiatric conditions	Digestive diseases	GAD	Gastritis	11.111	2.298	1.775	2.942
Digestive diseases	Psychiatric conditions	Gastritis	GAD	14.887	2.290	1.767	2.933
Psychiatric conditions	Cardiovascular diseases	Burnout	Hyperlipidemia	11.253	2.276	1.564	3.232
Musculoskeletal diseases	Psychiatric conditions	Sciatica	GAD	8.120	2.264	1.616	3.109
Psychiatric conditions	Neurological diseases	Depression	Bothersome tinnitus	20.199	2.240	1.606	3.076
Psychiatric conditions	Musculoskeletal diseases	Panic disorder	Sciatica	11.423	2.233	1.611	3.036
Urogenital diseases	Digestive diseases	Prostate problems	Gastric acid reflux	5.287	2.200	1.643	2.915
Psychiatric conditions	Cardiovascular diseases	Depression	Hyperlipidemia	16.880	2.184	1.592	2.952
Psychiatric conditions	Musculoskeletal diseases	Depression	Sciatica	18.727	2.158	1.668	2.765
Musculoskeletal diseases	Psychiatric conditions	Sciatica	Panic disorder	8.579	2.130	1.535	2.900
Digestive diseases	Musculoskeletal diseases	Gastritis	Sciatica	14.232	2.126	1.601	2.784
Cardiovascular diseases	Digestive diseases	Cardiac arrhythmia	Gastric acid reflux	4.586	2.123	1.562	2.846
Digestive diseases	Cardiovascular diseases	Gastric acid reflux	Cardiac arrhythmia	24.490	2.122	1.562	2.846
Neurological diseases	Psychiatric conditions	Bothersome tinnitus	Depression	4.502	2.120	1.520	2.910
Digestive diseases	Skin diseases	Gastric acid reflux	Acne	20.574	2.114	1.815	2.458
Skin diseases	Digestive diseases	Acne	Gastric acid reflux	21.465	2.106	1.808	2.448
Neurological diseases	Psychiatric conditions	Migraine	GAD	13.383	2.091	1.586	2.718
Psychiatric conditions	Cardiovascular diseases	Depression	Hypertension	17.028	2.090	1.603	2.699
Psychiatric conditions	Neurological diseases	Panic disorder	Migraine	11.150	2.089	1.595	2.700
Digestive diseases	Psychiatric conditions	Gastric acid reflux	Burnout	23.710	2.089	1.680	2.582
Psychiatric conditions	Digestive diseases	Depression	Gastric acid reflux	19.236	2.089	1.777	2.448
Psychiatric conditions	Digestive diseases	Burnout	Gastric acid reflux	9.363	2.088	1.680	2.579
Neurological diseases	Psychiatric conditions	Migraine	Panic disorder	13.361	2.087	1.593	2.698
Digestive diseases	Psychiatric conditions	Gastric acid reflux	Depression	22.288	2.083	1.772	2.442
Psychiatric conditions	Neurological diseases	GAD	Migraine	10.446	2.079	1.577	2.704
Skin diseases	Skin diseases	Herpes	Acne	23.321	2.079	1.801	2.394
Musculoskeletal diseases	Psychiatric conditions	Sciatica	Depression	7.380	2.069	1.599	2.652
Musculoskeletal diseases	Digestive diseases	Sciatica	Gastritis	8.530	2.061	1.551	2.701
Skin diseases	Skin diseases	Acne	Herpes	21.269	2.061	1.786	2.373
Psychiatric conditions	Cardiovascular diseases	Burnout	Hypertension	10.851	2.055	1.487	2.791
Cardiovascular diseases	Psychiatric conditions	Hyperlipidemia	Depression	4.871	2.020	1.474	2.727
Psychiatric conditions	Digestive diseases	GAD	Lactose intolerance	10.802	2.018	1.483	2.699
Digestive diseases	Psychiatric conditions	Lactose intolerance	GAD	10.526	2.006	1.474	2.683
Skin diseases	Digestive diseases	Herpes	Gastric acid reflux	24.076	1.992	1.724	2.297
Digestive diseases	Skin diseases	Gastric acid reflux	Herpes	21.047	1.990	1.723	2.295
Musculoskeletal diseases	Neurological diseases	Chronic back pain	Migraine	8.803	1.965	1.448	2.620
Neurological diseases	Musculoskeletal diseases	Migraine	Chronic back pain	12.953	1.964	1.447	2.618
Skin diseases	Urogenital diseases	Herpes	Prostate problems	21.053	1.954	1.446	2.611
Cardiovascular diseases	Psychiatric conditions	Hypertension	Depression	7.528	1.930	1.480	2.492
Digestive diseases	Musculoskeletal diseases	Gastric acid reflux	Chronic back pain	23.489	1.913	1.515	2.397
Musculoskeletal diseases	Digestive diseases	Chronic back pain	Gastric acid reflux	8.662	1.905	1.509	2.387
Skin diseases	Skin diseases	Eczema	Acne	21.429	1.871	1.609	2.170
Digestive diseases	Digestive diseases	Gastritis	Lactose intolerance	13.117	1.870	1.414	2.437
Skin diseases	Skin diseases	Acne	Eczema	22.006	1.868	1.607	2.166

Group of Condition 1	Group of Condition 2	Condition 1 …	… in individuals with Condition 2	Prevalence (%)	Odds ratio	Lower 95%-CI	Upper 95%-CI
Digestive diseases	Digestive diseases	Lactose intolerance	Gastritis	9.540	1.862	1.408	2.426
Digestive diseases	Neurological diseases	Gastric acid reflux	Migraine	21.244	1.822	1.491	2.213
Neurological diseases	Digestive diseases	Migraine	Gastric acid reflux	11.529	1.821	1.491	2.212
Digestive diseases	Skin diseases	Gastric acid reflux	Eczema	19.749	1.801	1.543	2.097
Skin diseases	Digestive diseases	Eczema	Gastric acid reflux	20.064	1.800	1.542	2.095
Digestive diseases	Cardiovascular diseases	Gastric acid reflux	Hypertension	20.367	1.773	1.395	2.236
Skin diseases	Digestive diseases	Herpes	Gastritis	23.232	1.736	1.444	2.079
Digestive diseases	Skin diseases	Gastritis	Herpes	11.526	1.736	1.443	2.078
Psychiatric conditions	Skin diseases	Depression	Acne	16.667	1.736	1.469	2.044
Cardiovascular diseases	Digestive diseases	Hypertension	Gastric acid reflux	7.771	1.712	1.349	2.158
Psychiatric conditions	Pulmonary diseases	Depression	Asthma	16.683	1.710	1.402	2.072
Digestive diseases	Skin diseases	Gastritis	Eczema	11.097	1.709	1.406	2.066
Skin diseases	Psychiatric conditions	Acne	Depression	20.148	1.708	1.445	2.012
Pulmonary diseases	Psychiatric conditions	Asthma	Depression	12.915	1.706	1.398	2.068
Skin diseases	Digestive diseases	Eczema	Gastritis	19.865	1.706	1.403	2.062
Digestive diseases	Skin diseases	Gastritis	Acne	9.890	1.664	1.361	2.021
Skin diseases	Digestive diseases	Acne	Gastritis	18.182	1.650	1.350	2.004
Pulmonary diseases	Digestive diseases	Asthma	Gastric acid reflux	11.975	1.548	1.281	1.860
Digestive diseases	Pulmonary diseases	Gastric acid reflux	Asthma	17.922	1.546	1.279	1.858
Skin diseases	Skin diseases	Herpes	Eczema	20.564	1.532	1.319	1.774
Skin diseases	Skin diseases	Eczema	Herpes	18.263	1.530	1.317	1.772

Abbreviations: CI = Confidence Interval; GAD = Generalized Anxiety Disorder; IBS = Irritable Bowel Syndrome; OCD = Obsessive-Compulsive Disorder; PTSD = Posttraumatic Stress Disorder; UTI = Urinary Tract Infection.
